# Multivariate transcriptome analysis identifies networks and key drivers of chronic lymphocytic leukemia relapse risk and patient survival

**DOI:** 10.1186/s12920-021-01012-y

**Published:** 2021-06-29

**Authors:** Ti’ara L. Griffen, Eric B. Dammer, Courtney D. Dill, Kaylin M. Carey, Corey D. Young, Sha’Kayla K. Nunez, Adaugo Q. Ohandjo, Steven M. Kornblau, James W. Lillard

**Affiliations:** 1 Department of Microbiology, Biochemistry, and Immunology, Morehouse School of Medicine, 720 Westview Dr SW, HG 341B, Atlanta, GA 30310 USA; 2 Department of Biochemistry, Emory University School of Medicine, Atlanta, GA 30322 USA; 3 Department of Leukemia, University of Texas MD Anderson Cancer Center, Houston, TX 77030 USA

**Keywords:** CLL, Networks, Relapse, Survival, WGCNA, ARHGAP27P2, C1S, CASC2, CLEC3B, CRY1, CXCR5, FUT5, MID1IP1, URAHP

## Abstract

**Background:**

Chronic lymphocytic leukemia (CLL) is an indolent heme malignancy characterized by the accumulation of CD5^+^ CD19^+^ B cells and episodes of relapse. The biological signaling that influence episodes of relapse in CLL are not fully described. Here, we identify gene networks associated with CLL relapse and survival risk*.*

**Methods:**

Networks were investigated by using a novel weighted gene network co-expression analysis method and examining overrepresentation of upstream regulators and signaling pathways within co-expressed transcriptome modules across clinically annotated transcriptomes from CLL patients (N = 203). Gene Ontology analysis was used to identify biological functions overrepresented in each module. Differential Expression of modules and individual genes was assessed using an ANOVA (Binet Stage A and B relapsed patients) or T-test (SF3B1 mutations). The clinical relevance of biomarker candidates was evaluated using log-rank Kaplan Meier (survival and relapse interval) and ROC tests.

**Results:**

Eight distinct modules (M2, M3, M4, M7, M9, M10, M11, M13) were significantly correlated with relapse and differentially expressed between relapsed and non-relapsed Binet Stage A CLL patients. The biological functions of modules positively correlated with relapse were carbohydrate and mRNA metabolism, whereas negatively correlated modules to relapse were protein translation associated. Additionally, M1, M3, M7, and M13 modules negatively correlated with overall survival. CLL biomarkers BTK, BCL2, and TP53 were co-expressed, while unmutated IGHV biomarker ZAP70 and cell survival-associated NOTCH1 were co-expressed in modules positively correlated with relapse and negatively correlated with survival days.

**Conclusions:**

This study provides novel insights into CLL relapse biology and pathways associated with known and novel biomarkers for relapse and overall survival. The modules associated with relapse and overall survival represented both known and novel pathways associated with CLL pathogenesis and can be a resource for the CLL research community. The hub genes of these modules, e.g., ARHGAP27P2, C1S, CASC2, CLEC3B, CRY1, CXCR5, FUT5, MID1IP1, and URAHP, can be studied further as new therapeutic targets or clinical markers to predict CLL patient outcomes.

**Supplementary Information:**

The online version contains supplementary material available at 10.1186/s12920-021-01012-y.

## Background

Chronic lymphocytic leukemia (CLL) is a heme malignancy characterized by the presence of CD5^+^ CD19^+^ B cells in the blood, bone marrow, and lymph node organs [1]. In 2020, CLL was predicted to occur in 21,040 new cases and lead to 4060 deaths, in the United States [2]. Recent therapy innovations have markedly improved response rates, and the duration of response for most, but patients continue to develop resistance to therapy, resulting in relapse. CLL does not have a cure and is heterogeneous in terms of progression and outcomes. The molecular mechanisms responsible for relapse are incompletely understood. Patient survival can range from a few months to several years [3]. The aggregation of B cells in the bone marrow and lymphoid organs interferes with the production of new blood, resulting in anemia, thrombocytopenia and neutropenia as well as impairing immune system integrity negatively impacting the quality of life of CLL patients. To effectively cure CLL and improve the quality of life of CLL patients, we need a better understanding of the cellular and molecular mechanisms that follow CLL initiation and lead to disease progression.

Many molecular features and biomarkers have been identified for CLL that drive disease progression or clinically prognostic. Numerous cytogenetic abnormalities with favorable and unfavorable prognostic impact are recognized. The five major mutations in CLL include the 13q (mir-15), 11q (ATM), 17p (TP53), and 6q (FOXO) deletions and Trisomy 12 (NOTCH1) [4]. The 17p, 11q, 6q, and Trisomy 12 mutations result in lower overall survival, time to first treatment, and progression free survival [4]. In the current targeted therapy era (i.e., Ibrutinib, Venetoclax), the 17p deletion is the only cytogenetic aberration used to inform treatment decisions [5]. The Ig heavy chain variable region's mutation status is prognostic with unmutated forms (U-CLL) showing enhanced B cell receptor (BCR)-signaling and being prognostically adverse in patients treated with chemotherapy [6–8]. Mutated CLL (M-CLL) cells possess a rearranged IGHV, are derived from B cells that have undergone somatic hypermutation, and have decreased BCR-signaling, which results in a more indolent disease [6]. Some cytogenetic abnormalities are more prevalent based on IGHV status (i.e., Trisomy 12) which explains distinct differences in U and M-CLL biology and outcomes [9, 10]

Although not mutated, upregulation of BCR, PI3K and BCL-2 anti-apoptotic molecules are involved in signaling pathways that drive CLL progression [11] and activate downstream effectors, e.g., JNK, ERK, mTOR, and NF-kB, which promote anti-apoptotic effects, growth, and proliferation [11–13]. These pathways are targets for therapies, e.g., BCL-2 and BTK inhibitors, venetoclax and ibrutinib, respectively [14, 15]. Even with these effective targeted therapies, CLL patients often relapse.

Relapse is a common occurrence during CLL treatment. When patients are treated with the standard fludarabine, cyclophosphamide, and rituximab (FCR) therapy, 6% of patients experience relapse within 6–12 months, and 14% within 2 years [16, 17]. The 5-year progression free survival rate of refractory/relapsed CLL patients treated with ibrutinib is 44% [18]. Previously reported factors contributing to relapse are aberrant expression of BTK (ibrutinib), and BCL-2 (venetoclax). In 17p deletion relapse cases, 80% of patients developed mutations in BTK, or PLCγ2, which cause ibrutinib to become ineffective [19]. Apart from these findings, mechanisms responsible for relapse have not been completely characterized.

This study aimed to identify the gene expression patterns associated with clinical outcomes. This is the first study to our knowledge to use a novel WGCNA, a systems biology method, to determine how these molecular signatures, across the transcriptome network, are associated with the clinical attributes of CLL. Furthermore, this study aims to contribute to understanding the biology of this disease.

## Methods

### Reads per kilobase of transcript per million mapped Reads (RPKM) and clinical data

CLL patient RNA-seq RPKM and clinical data were downloaded from the ICGC CLLE-ES project [20]. RNA-seq expression data was sequenced via an Illumina Hiseq 2000 sequencer and aligned with the human reference genome Gencode v7 hg19. Gene counts were estimated using a transcriptome counter program called Flux Capacitor. An overview of clinical data for 203 case samples is provided (Table 1).

**Table 1:** Distribution of reported clinical traits among CLL patients

Clinical traits	Description	Number of patients with reported information
Age at diagnosis	Age patient was when diagnosed with disease	203
Survival days	Length of time patient survived (days) since primary diagnosis	203
Binet stage B	Patients diagnosed with Binet Stage B CLL	15/203
Binet stage A	Patients diagnosed with Binet Stage A CLL	179/203
Binet stage C	Patients diagnosed with Binet Stage C CLL	8/203
Male		116/203
Female		78/203
Relapse interval	Length of disease-free interval (days) following primary treatment	92/203
Relapse	Patients with reported relapse event	92/203
Chemotherapy	Patients who received chemotherapy as first treatment	24/203
No treatment	Patients who did not receive any therapy	9/203
IGHV mutated	Patients with Immunoglobulin Heavy Chain mutations	132/203
IGHV unmutated	Patients without Immunoglobulin Heavy Chain mutations	65/203
SF3B1 mutated	Patients with any SNP mutations in the SF3B1 gene	19/203
ATM mutated	Patients with any mutations in the ATM gene	18/203

These clinical traits were correlated with transcriptome module eigengenes that were identified during the Weighted Gene Co-Expression Network Analysis (WGCNA)

**Fig. 1:**
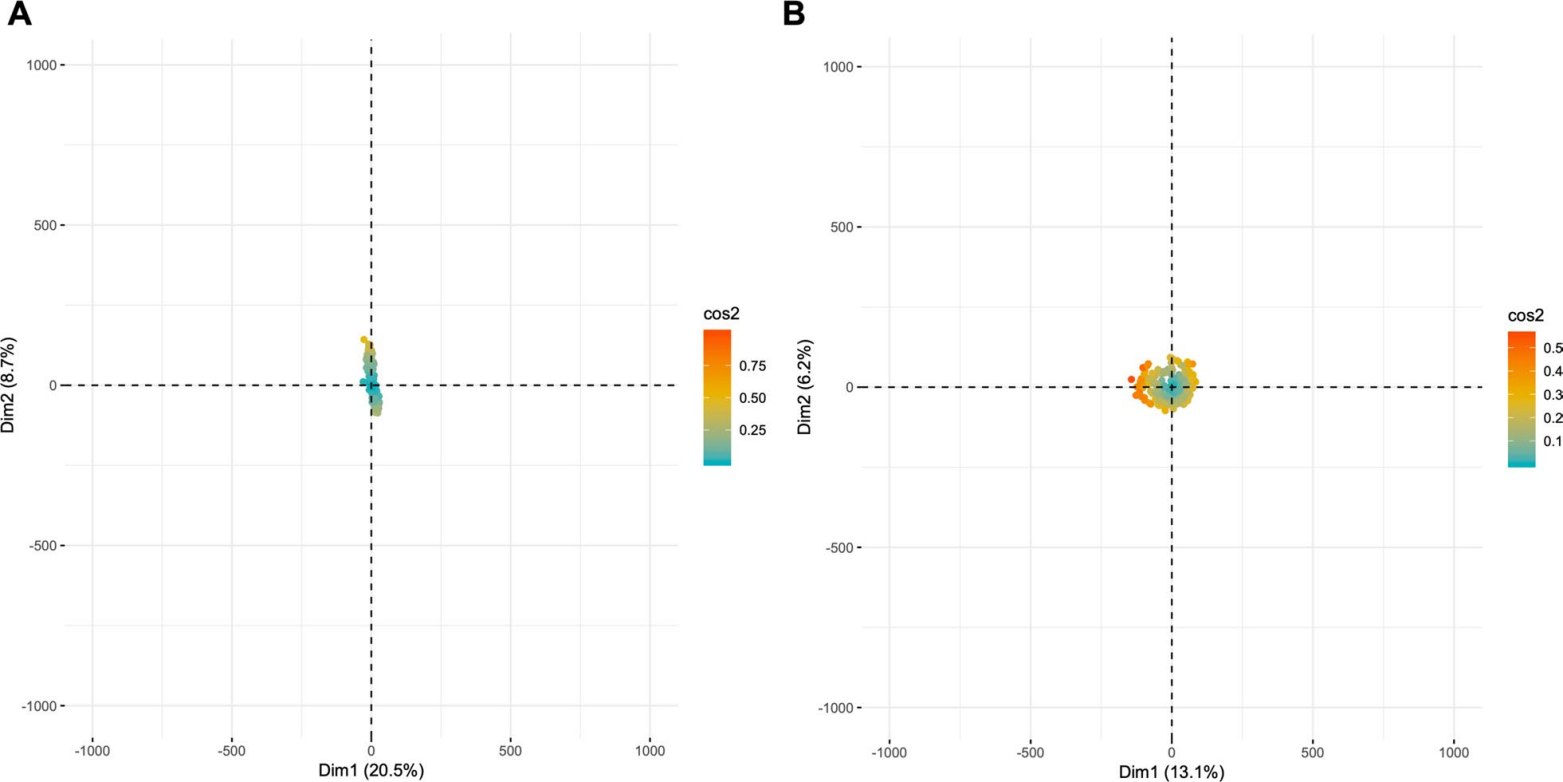
Principle component analysis (PCA) of CLL patient RNA-seq data before and after data cleaning steps. **A** The variation in the dataset before data cleaning and **B** represents the variation in the dataset after the data cleaning steps. The x and y axes illustrate the variation between the samples

Data cleaning was performed to reduce variation in the gene set, of 57,820 genes, before gene clustering analysis. This was completed by removing genes with zero RPKM values in 50% or more of the patient samples. The remaining 24,658 genes were then log_2_ transformed following addition of 0.05 RPKM. Next, two outlier patients, with z-transformed sample connectivity (z.k.) measures that were three or more standard deviations from the average z.k of the patient cohort were removed iteratively until no such outliers were detected before gene clustering. A principal component analysis was performed, using the R statistical program and Factoextra package, to assess the quality of the dataset before and after data cleaning (Fig. 1). Variance in gene expression introduced by confounding variables (sex and age), was assessed using the VariancePartitian R package.

### Gene clustering

The WGCNA R package was used to identify gene co-expression networks and correlate them with several clinical traits and characteristics. WGCNA identifies gene clusters by creating a sample dissimilarity matrix (1-topology overlap) and grouping genes that have similar expression patterns within the patient cohort. The network construction was performed using WGCNA blockwiseModules function with parameters as follows: bicor correlation, signed network module separation, maxBlocksize of 25,000, and a power achieving scale-free topology of 5.5. We used “bicor”, biweight midcorrelation, as opposed to Pearson correlation, to robustly correlate with less weight given to outlier measures [21, 22]. Due to high technical variation within RNAseq data, representing transcript abundances across samples, bicorrho and *p* values were used to summarize correlation with more robustness, a pivotal feature of the analysis. The first principal component of each module (module eigengenes) was then correlated with clinical traits (Sex, deceased, alive, relapsed, survival days, age at diagnosis, IGHV status, SF3B1 mutated, ATM mutated, remission, stable, relapse interval, Binet Stage A, and Binet Stage B). Modules for Unmutated and Mutated CLL patients were built using the same parameters except for use of different optimum soft thresholding powers (3.5 for M-CLL, 6 for U-CLL). Variance in introduced by confounding variables (sex and age), in individual and module gene expression, was assessed using the VariancePartitian R package.

### Differential expression, ROC curve, and Kaplan–Meier analyses

Differential Expression via an ANOVA test was performed to identify gene biomarker candidates within modules over-expressed/downregulated in relapsed CLL patients. A one-way ANOVA was used to compare Binet Stage A CLL patients who relapsed (n = 78) with those that did not relapse (n = 102). Of the patients who relapsed, 22 received chemotherapy and 17 received chemotherapy with an anti-CD52 antibody. The remaining patients had unreported therapy information. As the sequencing data for the CLL-ES project was produced prior to the FDA approval of Ibrutinib and Venetoclax, the therapy options for these CLL patients were chemotherapy (fludarabine, cyclophosphamide, bendamustine, chlorambucil) combined with an antibody (rituximab and alemtuzumab). 18,044 genes with symbols were visualized using the EnhancedVolcano R package and used for further analyses. To determine if differentially expressed genes could serve as biomarkers for relapse, a Receiver Operator Curve Analysis (ROC) was performed using the expression of the top over-expressed genes in relapse-associated modules (M4, M13, M10, M3, and M7). This analysis was performed using the easyROC web tool on the default non-parametric test setting. The control group were patients who did not experience a relapse event (n = 108). Cutoff points for overall survival and relapse free survival were calculated for differentially expressed hub genes with symbols (kME of 0.7 and log fold change of 0.5 and above) from networks correlated with survival days and relapse, using the cutpointr R package. For the gene combination ROC analysis, z-transformed gene expression data was used as input to calculate prognostic ratio scores, per patient, for combinations (pairs and triplets) generated from the aforementioned candidate biomarkers and genes from modules (M2 and M11) down-regulated in relapsed patients. Prognostic ratios were calculated by dividing the expression of over-expressed genes by the expression of down-regulated genes to amplify sensitivity for relapse prediction which is denoted by changes in a sample. This analysis generated 71,359 combinations to be tested at four relapse interval time points (15 months, 18 months, 3 years, and 5 years) with the pROC R package.

### Gene ontology analysis

GO Elite was used to perform a gene ontology enrichment analysis on gene symbol lists from transcripts within modules of interest to identify their functions [23]. Gene enrichment analysis involves using predefined lists classifying genes of interest into categories, such as biological processes and molecular functions, and testing for statistical overrepresentation of the category members, in this case, to gene lists based on module membership. Fisher’s exact test was used to test for over-representation or significant list overlap [24]. In addition to the standard Ensemble v6.2 database with 3 standard ontology categories, the Gene Set Enrichment Analysis (GSEA) molecular signature C2 database (v6.2) was used as a reference to identify association of network modules with the curated lists related to published studies with varying focus on health and disease, particularly cancer-dysregulated gene lists [25, 26].

### Module preservation

WGCNA’s modulePreservation function was used to test whether gene correlations within the ICGC modules exist in a separate cohort. The Broad CLL RNA-seq dataset (n = 93, 17,000 genes) was used as the validation set. ICGC samples in the Broad study were removed prior to data processing. The validation dataset was processed in the same manner as the ICGC dataset: outlier patient samples and genes with low expression were removed and the dataset was log2 transformed prior to module preservation analysis. The module preservation function was also used to assess preservation of modules between regressed and unregressed ICGC data and mutated (n = 132) and unmutated ICGC CLL patients (n = 65).

## Results

### Systems biology defines a network of CLL co-expression modules

**Fig. 2:**
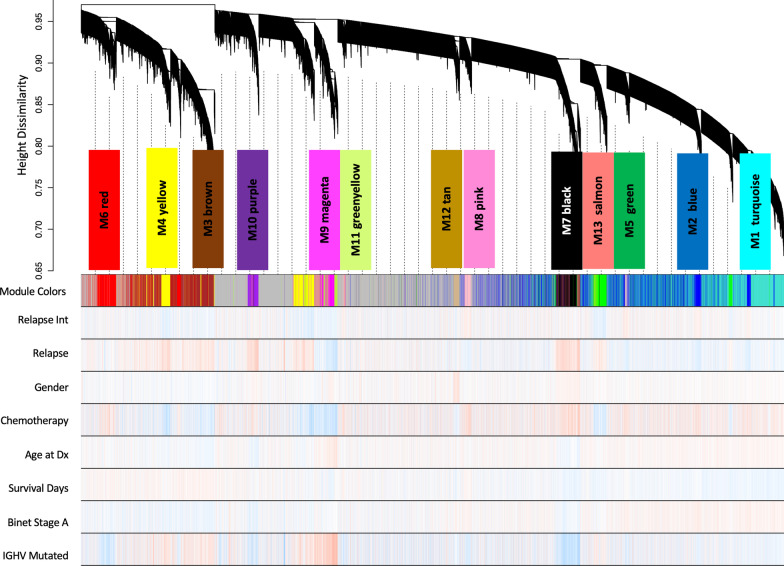
Cluster dendrogram of module eigengenes detected based on dissimilarity in WGCNA. There were 13 modules identified within the CLL patient gene expression data. WGCNA determined dissimilarities between modules by calculating correlations of gene expression patterns across all patient samples and clinical traits clinical traits (i.e., Relapse Interval, Relapse, Gender, Chemotherapy, Age at Diagnosis, Survival Time in Days, Binet Stage A, and IGHV Mutated)

**Fig. 3:**
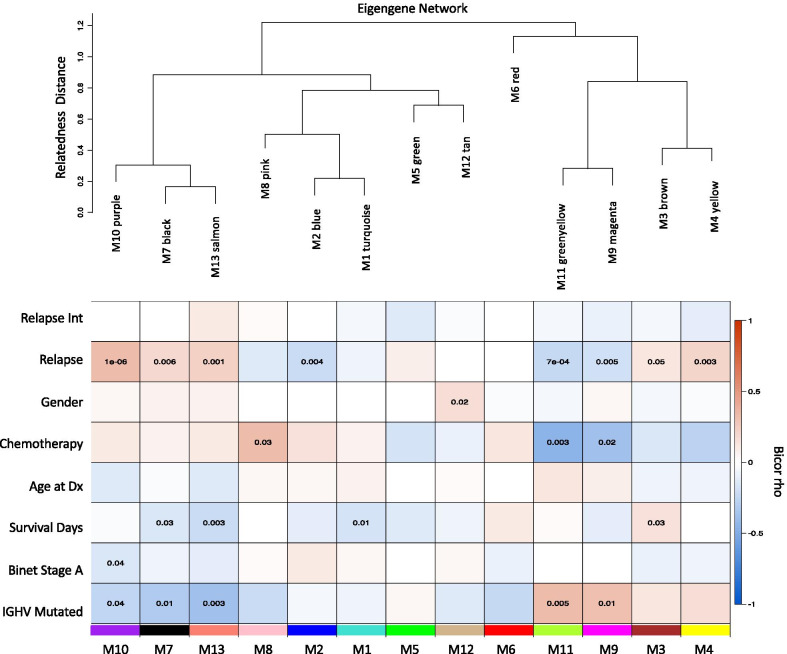
Module eigengene heatmap with clinical correlates. Upper panel, Module eigengene relatedness dendrogram based on the correlation network of MEs. Lower panel. The colors (bottom) represent different co-expressed gene clusters in order of relatedness. The heatmap color scale is for bicor rho from − 1 (anti-correlated) to + 1, (correlated) Correlations of the module eigengenes to clinical traits (i.e., Relapse Interval, Relapse, Gender, Chemotherapy, Age at Diagnosis, Survival Time in Days, Binet Stage A, and IGHV mutated). Significant student correlation *p* values are given within the heatmap

To assess the systems biology of CLL, the transcriptome comprising 24,658 genes across 201 CLL case samples (Table 1) were examined for co-expression modules of gene transcripts. Thirteen modules eigengenes (MEs), numbered by their rank from the largest number of genes to the smallest, M1 to M13, were identified (Fig. 2; Table 2). After MEs were identified, their relatedness was determined by an expression correlation metric and plotted as a dendrogram (Fig. 3, upper panel). M10 was closely related to M7 and M13. M8, M2, and M1 were separate but also closely related, as were M5 and M12. The relatedness dendrogram showed M6, M11, and M9 formed a branch, as do M3 and M4 modules. Next, we asked whether the ICGC intramodule gene correlations were reproducible. This question was addressed using WGCNA’s modulePreservation function, the ICGC modules/dataset as a reference, and RNA-seq data from a Broad CLL study as the validation set [27]. Correlations in ten (i.e., blue, black, brown, green, greenyellow, magenta, purple, red, and turquoise) of the thirteen networks were preserved in the Broad dataset (Additional file 1).

**Table 2:** Gene networks and their number of genes

Module number	Color	Size
M1	Turquoise	4223
M2	Blue	3156
M3	Brown	1937
M4	Yellow	1315
M5	Green	1300
M6	Red	1260
M7	Black	990
M8	Pink	626
M9	Magenta	525
M10	Purple	479
M11	Greenyellow	424
M12	Tan	317
M13	Salmon	207

Networks number and colors are displayed from the largest to smallest size

### Transcript modules associated with relapse

Association of MEs to quantitative and qualitative clinical traits is assessed by correlation, reducing the multiple testing problem. This allows the determination of which modules are candidates for molecular causality of the traits of interest in this CLL cohort (Table 1). To determine the transcriptome networks of interest associated to case-sample traits, robust correlation of the 13 MEs to 8 traits including relapse status and survival time was performed (Fig. 3, heatmap). We identified M10 (*p* = 1E−08, R = 0.34), M7 (*p* = 0.008, R = 0.2), M13 (*p* = 0.001, R = 0.23), M3 (*p* = 0.05, R = 0.14), and M4 (*p* = 0.003, R = 0.21) as positively associated with relapse, and M2 (*p* = 0.004, R = −0.2), M11 (*p* = 7E−04, R = −0.24), and M9 (*p* = 0.005, R = −0.19) showed an opposing pattern anticorrelated to relapse status, a total of eight modules (Fig. 3, heatmap). Of these, M3 (*p* = 0.03, R = 0.16) positively associated with survival time, while M7 (*p* = 0.03, R = −0.15) and M13 (*p* = 0.003, R = −0.21) associated negatively. After observing many significant module-trait correlations, we endeavored to assess if any of the correlations may be due to confounding variables (sex and age). The variance introduced into gene expression, by sex, age, and IGHV status, was assessed using the Variance Partition R package. We observed that age and sex contribute to less than 10% of the variation observed in expression of any individual gene expression (Additional file 2, A) and most modules (Additional file 2, B). However, IGHV status contributed to 7–14% of the variance in expression of 5 modules: purple (M10), black (M7), salmon (M13), green (M5), and yellow (M4). Interestingly, the top 3 modules displaying some covariance to IGHV status (M10, M7, and M13) were also the most correlated with sex and age. However, these levels of covariance with all 3 variables were a minor component of the overall variance even in these modules.

**Fig. 4:**
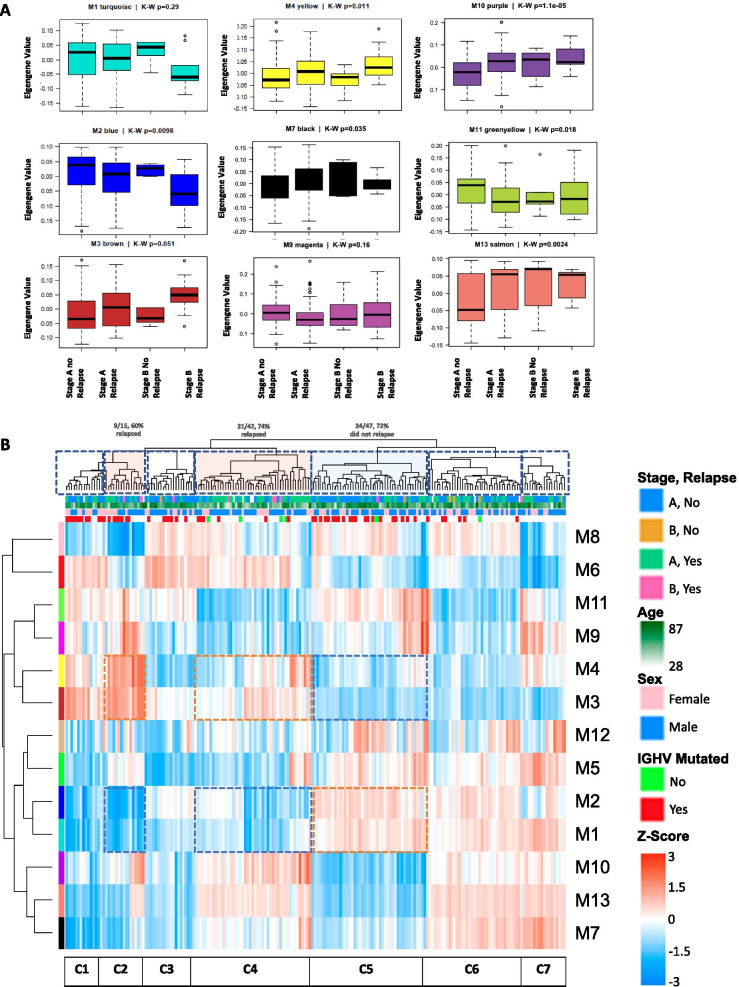
Differential expression patterns of module eigengenes across CLL patient samples. **A** Select ME boxplots and non-parametric ANOVA p for relapse status also segregated by CLL stage. **B** Clustering of MEs (rows) and samples (narrow columns) reveal a sample group (C4) that is enriched > 80% for relapsed individuals, and on average, has elevated expression of the yellow and brown module eigengenes, but downregulated expression of turquoise and blue module eigengenes. The opposite is apparent for the right cluster(C5) of case samples which have < 20% relapsed cases (13/47). The kME table hubs for these 4 modules confirms that these 2 sets of modules have the strongest correlation and anti-correlation in their pattern of expression across all 201 case-samples in the network. Color scale: eigengene expression Z-score

To decipher module-trait associations with relapse and overall survival, we performed a one-way ANOVA to compare the expression of eight relapse-associated modules and one patient survival-associated module between four groups: Binet stage A without relapse, Binet stage A with relapse, Binet stage B without relapse and Binet stage B with relapse. Seven modules were significantly differentially expressed between one or more of the comparison groups (Fig. 4A). To identify clusters of patients that exhibited relapse associated expression patterns of modules, we performed unsupervised hierarchical clustering of all 201 case-samples expression patterns of the 13 MEs (Fig. 4B). Seven patient clusters were formed (C1–C7). Interestingly, two patient clusters (C2 and C4) predominately experienced relapse events (dashed boxes filled with red color over sample dendrogram in Fig. 4B dendrogram). One cluster (C5) displayed the opposite composition, as it predominantly consisted of patients who did not experience a relapse event (dashed boxes with blue color over sample dendrogram in Fig. 4B). These patient clusters are of interest as they are possibly driving the expression patterns influencing the WGCNA module trait heatmap correlations and can be used to determine which patients are at risk for relapse. In the ME-sample heatmap, clusters C2 and C4 have above average expression of genes in the M3 and M4 (orange dashed boxes) modules and below average expression of M1 and M2 (blue dashed boxes). Cluster C5 has inversed expression patterns as the majority of this group consists of patients who did not experience a relapse event. Thus, the expression patterns of M1–M4 are associated with relapse status.

We next assessed the relationship between M1 and M2 with M3 and M4 expression modules transcripts, using Pearson correlations to MEs (kMEs). When kME is positive and above 0.7, and higher for one module than others, this identifies a gene as representative of the ME and is consistent with that module's membership. Such genes are considered as network hubs in a signed co-expression network. Hubs tend to be the network's key drivers. In co-expression analysis, the directionality of cause-and-effect relationship is not determinable without external information [28, 29]. The correlation table (Additional file 3) was sorted for ranked hubs (highest-to-lowest kME transcripts within their assigned module by WGCNA clustering), and correlation coefficients were colored by a red-yellow-green heatmap scale to represent positive correlation (red) and negative correlation (green) patterns across modules. Strikingly, M1 and M2 hubs shared positive correlation to each other, and negative correlation to M3 and M4 hubs. M3 and M4 hubs were inverted in their pattern. Thus, the overall expression patterns of these modules are interlinked.

### Transcript modules associated with relapse stratified by IGHV status

Previously, we observed IGHV status was a modest contributor to variance observed in several modules that were correlated with relapse. This implicated that modules associated with relapse risk may be influenced by or depend on IGHV status. To further explore this relationship, patients were separated by IGHV status and transcriptomes were analyzed separately for coexpression. The U-CLL group (n = 65) produced 17 modules (Additional file 4, A) whereas the M-CLL group (n = 132) produced 12 (Additional file 4, B). After identifying modules in both sets, we asked if IGHV status alters co-expression network structure or not using module preservation. Modules in the network with gene expression were regressed to remove IGHV covariance and were entirely and highly preserved in the original network and vice versa (Additional file 5, A and B). Regressed network modules and their module-trait relationships can be found in Additional file 6 (B, C). Moreover, we successfully cross-validated the preservation of both sets of modules in the separate M and U-CLL networks without regression (Additional file 5, C and D), implicating that observed gene correlation patterns are retained irrespective of IGHV status. None of the modules in the U-CLL group correlated with relapse, whereas 4 (Mut-M6, Mut-M10-12) correlated with relapse (Additional file 4). We followed up with a Wilcoxon t-test between Relapsed Binet Stage A and Non-relapsed Binet Stage A patients to determine if there were significant increases/decreases in expression of modules associated with relapse. To our surprise, the unmutated IGHV brown network module (U-M3) gene expression was increased in relapsed U-CLL patients (Additional file 7, B). Similar differences in expression were observed with M-CLL’s relapse associated modules (Additional file 8, A).

Gene ontology analysis of relapse-associated modules in M-CLL revealed translation (Mut-M6), cell growth, transcription, metabolism (Mut-M10), Rho protein signal transduction (Mut-M11), chromatin organization, homeostasis, and gene expression (Mut-M12) biological functions (Additional file 9). The U-M3 module in U-CLL had similar biological functions (macromolecule metabolism, mRNA processing) in addition to RNA splicing (Additional file 10). These results suggest that relapsed U-CLL patients exhibit increased levels of expression of genes involved in RNA splicing compared to U-CLL patients that did not experience relapse; relapsing M-CLL patients also showed an increase in translation machinery transcripts.

### Networks associated with known drivers of CLL

We next explored co-expression relationships of genes that have been previously shown to drive CLL progression. BTK, TP53, and BCL2 are co-expressed with genes in the M1 module, which was negatively correlated with survival days (*p* = 0.01, R = −0.18; Additional file 3). ATM was co-expressed with 3156 genes in the M2 module. ZAP70 and NOTCH1 were in the M7 and M13 modules. CXCR5 is co-expressed with genes in the M4 module. CD38, CXCL13, MCL1, and SF3B1 were not co-expressed with any genes. We noted CXCL13, CD38, MCL1, and SF3B1 did not fall into a module due to the lack of strong co-expression with over 30 gene products. However, we identified modules associated with the SF3B1 mutation status in all CLL and separately U and M-CLL patient networks. From the analysis of all CLL patients, the purple (M10) and greenyellow (M11) correlated with SF3B1 mutation status. Modules Mut-M4, Mut-M10. U-M6, and U-M10 were negatively correlated with SF3B1 mutation status. A Wilcoxon test was used to confirm decreased expression of Mut-M4 (Additional file 8, B) and U-M6 (Additional file 7, A). In U-CLL, our gene ontology results for enriched processes in U-M6 imply that SF3B1 mutations are associated with decreased proteolysis, organelle organization, mRNA transport, cell cycle regulation, and ARF protein signaling (Additional file 10). Defining overrepresented ontologies of the 13 modules’ hubs (Table 3) revealed that the key networks M1 through M4 represent ontologies including carbohydrate metabolic process (M1), mRNA metabolic process (M3), and cellular metabolic process (M4). The M2, M13, M10, M7, and M11 modules did not have any significantly overrepresented biological processes. The M9 module was overrepresented with translation elongation and termination and viral transcription and infection processes. Given that M4 hubs overrepresent the proteasome (PSMB2, UBE2B, and UBAP1), we concluded that M4 is potentially relevant to upregulated proteostasis capacity of B cells in relapsed CLL.

**Table 3:** Functions for relapse associated networks

Module	Top 5 biological processes	FET *p* value
*Positively correlated with relapse*		
Purple	No significant biological processes identified	NA
*p* < 0.001		
Rho = 0.34		
Salmon	No significant biological processes identified	NA
*p* = 0.001		
Rho = 0.23		
Yellow	Cellular macromolecule metabolic process	2.90E−19
*p* = 0.003	Proteasomal protein catabolic process	2.27E−10
Rho = 0.21	Establishment of protein localization	5.11E−08
	Intracellular transport	4.14E−07
	Positive regulation of cell cycle arrest	1.29E−06
Black	No significant biological processes identified	NA
*p* = 0.006		
Rho = 0.19		
Brown	mRNA metabolic process	8.10E−11
*p* = 0.05	Regulation of macromolecule metabolism	1.24E−09
Rho = 0.14	Modification-dependent macromolecule catabolic process	3.51E−09
	RNA splicing regulation	5.81E−09
	Cellular metabolism process	1.91E−08
*Negatively correlated with relapse*		
Greenyellow	No significant biological processes identified	NA
*p* < 0.001		
Rho = −0.24		
Blue	No significant biological processes identified	NA
*p* = 0.004		
Rho = 0.2		
Magenta	Translational elongation	1.86E−110
*p* = 0.005	Viral transcription	1.17E−108
Rho = −0.2	Translational termination	1.37E−106
	Viral infectious cycle	1.89E−106
	Endocrine pancreas development	1.76E−103

Networks adjacent to the red bar have positive correlations with relapse and are upregulated in Binet Stage A patients who relapsed. Networks adjacent to the blue panel are negatively correlated with relapse. The top five biological functions and adjusted *p* values are displayed in the table. Networks with blank biological processes did not have any overrepresented biological processes based on the FET

**Fig. 5:**
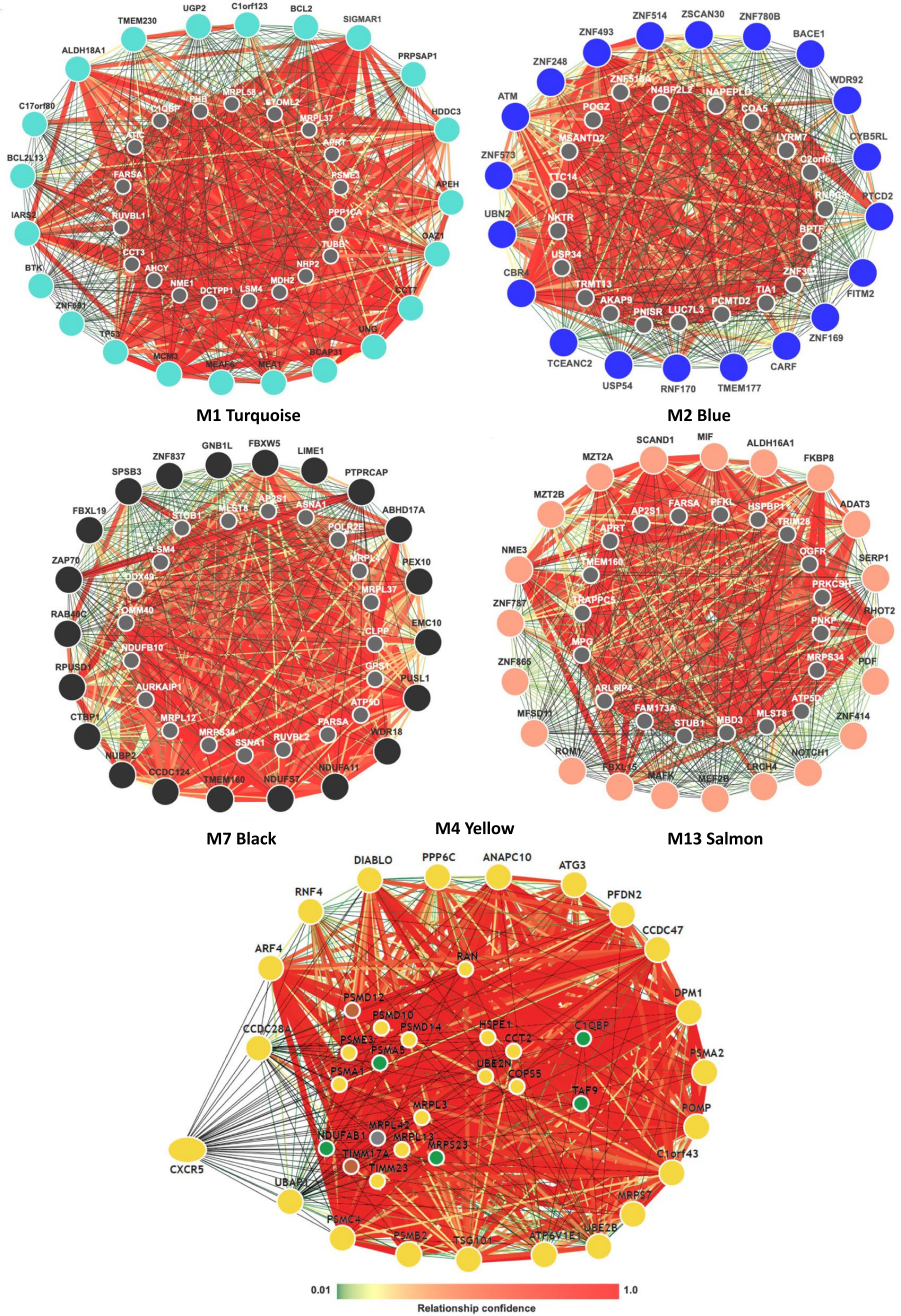
Predicted interactions between CLL Biomarkers and hub genes in their assigned modules. The hub genes are represented by the large nodes. The smaller nodes represent the additional genes added based on signaling biology reported in the literature. The heatmap scale represents the confidence of the predicted interactions based on the B cell specific interactions. Reded lines represent high confidence interactions

To identify B cell specific functional interactions between CLL biomarkers (BTK, TP53, BCL2, ZAP70, NOTCH1, CXCR5, and ATM) and hub genes of their assigned modules, we used Genome-wide Integrated Analysis of gene Networks in Tissues (GIANTv2) [30]. Deciphering these functional interactions are important, as they are implicative of mechanisms that promote CLL relapse. GIANTv2 calculates functional relationship Confidence Scores (CS) for each input gene interaction and all other gene interactions, based on what’s reported in the literature. TP53 has strong upstream interactions with M1 hub gene UNG (CS = 0.537), TUBB (CS = 0.73), PHB (CS = 0.87), MCM3 (CS = 0.95) and PPP1CA (CS = 0.97). BCL2 has high confidence upstream interactions with TP53 (CS = 0.744) and PPP1CA (CS = 0.34). ATM has upstream functional interactions with N4BP2L2 (CS = 0.89) and AKAP9 (CS = 0.86) in M3 brown. ATM has downstream functional interactions with USP34 (CS = 0.96), PNISR (CS = 0.94), TIA1 (CS = 0.91) in M1, NKTR (CS = 0.68), and LUC7L3 (CS = 0.57) in M1. ATM’s interactions implicate signaling communication between the M1, M2, and M3 modules, which was previously implicated in Fig. 4B. ZAP70 has a downstream interaction with PTPRCAP (CS = 1), a hub gene of the M7 black module. BTK, CXCR5, and NOTCH1 did not have any high confidence interactions with the top hub genes of their assigned modules respectively (Fig. 5). These results implicate that signaling relationships between BTK, CXCR5, NOTCH1 and their hub genes have not been characterized in the literature.

### Modules differentiating between relapsed and non-relapsed patients represent biological underpinnings of cancer

**Fig. 6:**
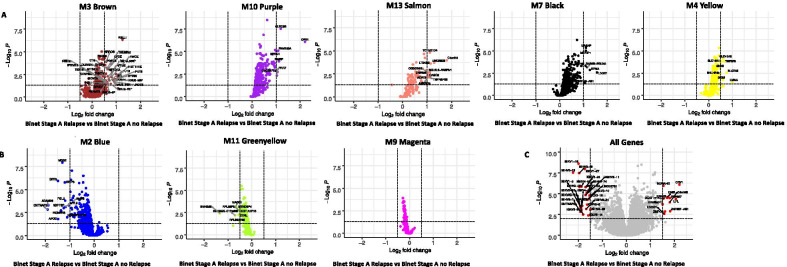
CLL relapse-correlated modules differentially expressed between CLL relapse versus patients who did not relapse. Differential expression was performed to compare Stage A CLL patients who relapsed (n = 78) with those that did not relapse (n = 102). **A** M3, M10, M13, M7, and M4 modules are biased towards genes upregulated in CLL patients that relapsed. **B** Illustrates how M2, M11, and M9 are biased towards downregulated genes in cases that relapsed. **C** Volcano plot of all 18,044 genes with symbols in the network. *P* values were calculated using ANOVA-Tukey

Based on the relapse trait correlations, we hypothesized that modules with positive correlations would have elevated expression and modules with negative correlations would be down regulated in patients who experience a relapse event. This hypothesis was tested using an ANOVA-Tukey analysis (Additional file 11). Consistent with the module-trait bicor correlations to relapse (Fig. 3, heatmap), the volcano plots for Binet Stage A relapsed versus Binet Stage A non-relapsed pairwise Tukey post hoc significance confirmed elevated expression of genes in M3, M4, M7, M10, and M13 (Fig. 6A) and lower expression of M2, M9, and M11 (Fig. 6B).

A total of 1703 genes were down regulated, and 1250 genes were upregulated between the Binet stage A relapse and Binet stage A no relapse groups (Additional file 11, *unfiltered full table*). Biological processes associated with the upregulated genes are negative regulation of monooxygenase activity, response to stimulus, cell communication regulation, cell signaling, and epithelial-to-mesenchymal transition. We noted that CLL biomarkers ATM, CXCR5, and ZAP70 were significantly differentially expressed. However, there was no difference in expression detected for BTK, BCL2, CD38, MCL1, NOTCH1, SF3B1, and TP53. Additionally, we noted the top 5 differentially expressed genes in each relapse-associated module (Fig. 6): CRY1,CLEC3B, RMRP, FAM166A, and PRR7 (M10 purple), C4ORF48, UNC93B2, LTBR2, TNFRSF25, and TCETX1D4 (M13 salmon), LMNA, SLC7A5, TNFSF9, SLC41A2, and CKS2 (M4 yellow), RELL1, ID1, HIST1H1E, DUSP4, FUT5, and RNU2-64P(M3 brown), URAHP, MID1IPI, ZAP70, RAB4-EGLN2, ITPKA (M7 black), RPL21P75, EEF1A1P4, RPL21, RPL30, and NPC2 (M9 magenta), MACC1, MT9D6P4, SNHG25, and RNASEK-C17orf49 (M11 greenyellow), MDS2, DPF3, CRIP3, FGL2, and ADAM29 (M2 blue).

We hypothesized that the top differentially expressed genes of relapse associated modules could be used to predict relapse risk. Relapse risk was evaluated using a Receiver Operating Characteristc (ROC) analysis. Among those listed above, CRY1(0.73), URAHP (0.716), MID1IP1(0.708), and CLEC3B (0.706) had the highest AUC scores for prediction of relapse, considering the measure of relapse free survival (Table 4). Additionally, we tested the capability of gene expression combinations to predict relapse risk at four time points: 15 months, 18 months, 3 years, and 5 years (Additional files 12–15). The input genes for this analysis were the relapse biomarker candidates (Table 4) in addition to down-regulated (relapse) genes in the blue (CNTNAP2, ADMA29, DFF3, APOD, HOMER3) and greenyellow (SNHG25, MACC1) modules and non-coexpressed genes (LPL, ZNF667, DMD, KANK2). We performed this analysis in all CLL patients and U-CLL patients using the pROC R package. When considering all CLL patients, we found that SLC7A5 + URAHP + FAM166A/CNTNAP2 + HOMER3 + MACC1 (AUC 80.33%) is the best combination of genes considered for predicting relapse events within 15–18 months. ID1 + LPL + FUT5/SNHG25 + APOD + ADAM29, C1S + TCTEX1D4/CNTNAP2 + HOMER3 were the optimum combinations of candidates for predicting relapse within 3 (AUC 71.24%) and 5 years (AUC 74.6%), respectively. In U-CLL, the following combinations were capable of predicting RFS within 15 months, 18 months, 3 years and 5 years: CRY1 + RELL1/CNTNAP2 + HOMER3 (AUC 84.66%), SLC7A5 + URAHP + FAM166A/CNTNAP2 + HOMER3 + MACC1 (AUC 81.64%), CKS2 + FAM166A/HOMER3 + ADAM29 (AUC79.55%), and UNC93B2 + SLC7A5/APOD + ADAM29 (AUC 82.91%).

**Table 4:** Receiver operating curves (ROC) for genes overexpressed in relapse-associated modules

Gene	AUC	Z	*p* value	Module
URAHP	0.71558	5.95301	0	Black
MID1IP1	0.70859	5.62656	0	Black
ZAP70	0.66843	4.2768	2.00E−05	Black
RAB4B.EGLN2	0.6482	3.93621	8.00E−05	Black
ITPKA	0.64456	3.7544	0.00017	Black
LDOC1	0.62717	3.13477	0.00172	Black
RELL1	0.6954	5.3149	0	Brown
HIST1H1E	0.66089	4.18598	3.00E−05	Brown
JADE3	0.66024	4.13126	4.00E−05	Brown
ID1	0.65624	4.03103	6.00E−05	Brown
FUT5	0.63222	3.39698	0.00068	Brown
CRY1	0.73077	6.38277	0	Purple
CLEC3B	0.70679	5.69858	0	Purple
RMRP	0.68482	4.94943	0	Purple
FAM166A	0.68297	4.91336	0	Purple
PRR7	0.65664	4.03721	5.00E−05	Purple
TCTEX1D4	0.68681	5.03117	0	Salmon
UNC93B2	0.68412	4.97913	0	Salmon
LTB4R2	0.68167	4.81932	0	Salmon
C4orf48	0.67123	4.61744	0	Salmon
TNFRSF25	0.61798	3.04507	0.00233	Salmon
SLC41A2	0.67612	4.65439	0	Yellow
TNFSF9	0.66653	4.36275	1.00E−05	Yellow
SLC7A5	0.65574	4.04569	5.00E−05	Yellow
CKS2	0.61918	2.97625	0.00292	Yellow
LMNA	0.6013	2.4887	0.01282	Yellow

Table of genes, AUC scores, and *p* values from ROC analysis. The input genes had AUC scores above 0.5. URAHP (0.711), MID1IP1(0.693), and APBB2(0.691) have the highest AUC scores. *p* values of zero represent significance less than 10E−06

**Fig. 7:**
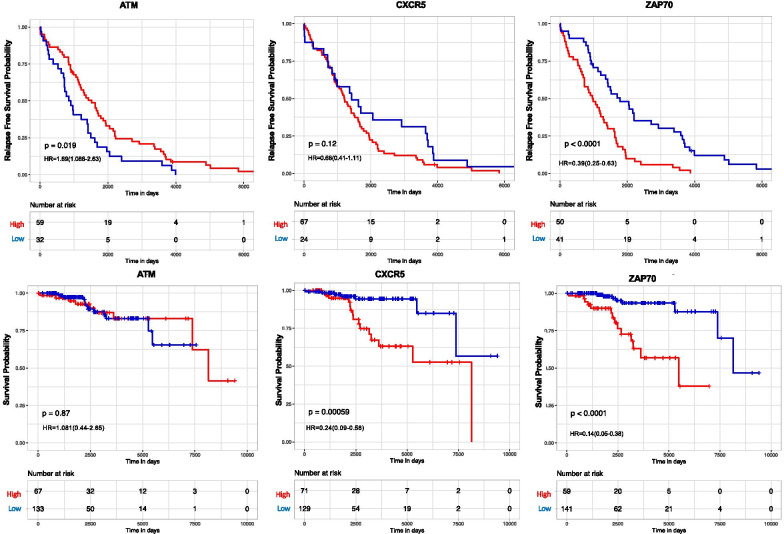
Overall and relapse free survival of biomarker genes. OS and RFS relationships for three biomarker genes (ATM, CXCR5, and ZAP70) from the M3, M4, M7, M10, and M13 networks were visualized. The red lines denote RFS of patients with high gene expression and the blue lines refer to patients with low expression. Hazard ratios (HR) are reported for low expression of the genes

To determine the effect of differentially expressed hub and biomarker genes on patient overall and relapse free survival outcomes, we performed a log-rank Kaplan Meier Analysis. Hub genes were selected for this analysis as they would make good therapeutic target candidates due to their capability to regulate the expression of multiple genes. Differentially expressed hub genes (*p* < 0.05, kME ≥ 0.7, LFC ≥ 0.5) from modules correlated with relapse (M3, M7, M10 and M13) and survival days (M3, M13, and M7) were evaluated (Additional file s 16 and 17). Hub genes from the M11, M9, M4, and M2 modules were not evaluated for RFS due to them not meeting the selection criterion. Hub genes from the M1 module were not evaluated for overall survival for the same reason. Hub genes ARHGAP27P2, HSPBP1, CASC2, and C1S along with CLL biomarkers CXCR5 and ZAP70 were prognostic for overall survival days (Additional file 16 and Fig. 7). Additionally, ATM, FUT5, and ZAP70 were prognostic for relapse free survival (Additional file 17 and Fig. 7).

## Discussion

Systems biology approaches can decipher complex interactions between cancer cell intracellular, extracellular, genetic, and epigenetic networks [31]. This study applied a systems biology approach to identify gene transcript co-expression networks, driven by CLL progression factors, and correlated them to clinical outcomes. The strongly correlated modules represented both known and novel mechanisms of heme malignancy.

A novel WGCNA method was used to identify modules of transcripts from CLL patient blood sample derived RNAseq data. Co-expression networks are mathematically defined without supervision and revealed sets of genes controlled by the same transcription factors or epigenetic regulation, have the same function, and therefore are co-regulated. Module members can also be enriched with genes of the same signaling pathway [32]. WGCNA identified 13 modules and correlated their expression with clinical traits.

Out of the thirteen modules, eight modules were found to be correlated with CLL relapse. Five had positive correlations (M10, M13, M4, M7, and M3) and three demonstrated negative correlations (M11, M2, and M9) (Table 3). These eight modules provide insights on pathways relapsed CLL cells depend on to proliferate and survive. Interestingly, the M11 and M9 modules contain genes involved in translation (Table 3). These modules positively correlated with the IGHV mutated disease status and negatively correlated with relapse and chemotherapy. Previously, it has been shown that CLL patients with high expression of ribosomal and translation associated proteins have lower progression free survival and reduced chemotherapy requirements [33]. In this study, we observed lower expression of translation associated proteins in patients (M9 and M11 in Fig. 6B) who experienced relapse events consistent with the poor clinical course reported in aforementioned study. Chemotherapeutic drugs often induce ROS-induced cell death. Higher expression of translation-associated proteins has been associated with oxidative stress induced apoptosis [34, 35]. Our study’s findings concur with previous findings that detail CLL cells with higher expression of translation-associated proteins may be more sensitive to chemotherapeutic drugs.

The M13 and M7 modules contain known drivers of CLL (NOTCH1 and ZAP70) and genes that can be used as predictors of relapse (CRY1, CLEC3B, MID1IP1, and URAHP). M13 and M7 also negatively correlate with survival days and the IGHV mutated disease status and positively correlated with relapse. High expression of ZAP70 is associated with poorer survival outcomes, increased BCR signaling, and the unmutated CLL disease status [36–38]. Although the prognostic value of ZAP70 expression has been examined, the molecular pathways its expression affects have not been completely characterized. In this study, we found that ZAP70 is co-expressed with genes involved in WNT (APC, AXIN1, DVL1, FZD2, STK11, WNT10A, and WNT6) and NOTCH (CFD, FURIN, HES7, MAML1, and MIB2) signaling and endocytosis (ABCA7, AP2A1, ARHGAP27, CORO1A, EPS15L1, HOOK2, LRP10, MARCH2, PDLIM7, RABEP2, RIN3, SCARF1, SCRIB, SH3GL1, SNX18, SPECC1L, UNC13D, and WASF2) and is significantly over-expressed in patients who relapsed (Additional file 8). Possibly, the M7 module associations are due to the fact that unmutated CLL cells have higher expression of WNT pathway genes and endocytose more antigens compared to mutated CLL cells [39]. In a subset of patients, NOTCH1 is constantly activated, often mutated, and is associated with ibrutinib resistance, poor clinical outcome and relapse [40–42]. Thus, supporting the M13 module’s negative correlation with days of survival [40, 43].

Interestingly, the M3 module displayed several genes of interest, associated with antigen presentation/receptor signaling (CD83), metabolism and apoptosis (CASP3, CASP8, RELA), DNA repair and gene instability (BRCA2, SUMO3, and KRAS), and GTP signaling/signal transduction (GNA13A and KRAS). In CLL, CD83 is overexpressed and has a role in creating an immunosuppressive environment [44–46]. DNA repair genes are also overexpressed and have been implicated in therapy resistance and relapse [47]. The M4 module contains genes involved in apoptosis (BCL2L11, BAK1, CASP7, DEDD2, DIABLO, FADD, TNF, and TNFRSF10B), the cell cycle (CDK7, CDK2, CDK9, CDK11B, and CDKN1B), and chemokine signaling (CXCR5, GNA12, RAC1, PIK3C3). During normal B cell maturation, CXCL13-CXCR5 signaling homes naïve B cells to bone marrow and lymphoid organs, at which point, the B cells undergo somatic hypermutation after antigen-specific BCR, SYK and LYN kinases are activated to promote the proliferation and survival of B cells [48]. Hence, co-expression of genes in this module increase the expression of pro-apoptotic proteins through the enhancement of NF-KB and PI3K signaling [49].

The CLL transcriptome co-expression network identified relationships between sets of MEs (Fig. 2). The genes in the M3 and M4 networks had an expression correlation rho above 0.5 and share the macromolecule metabolism biological function. Perhaps, the M3 module is closely related to the M4 module in function, because both share proteins that activate pathways represented by members of the cognate module. Previously, our laboratory treated prostate cancer cell lines with CXCL13 and observed an elevation in BRAC1, CTNNB1, ELK1, HDAC8, ICAM1, GNA13, MAPK8, RELA, RHOH, and STAT3 phosphorylation, all of which were present in the brown and yellow modules [50]. The phosphorylation of these proteins resulted in the growth, proliferation, survival, and metastasis of prostate cancer cells.

The M10 module has genes that are involved in B cell activation (CD28, IRS2, TGFB1, TICAM1, and TNFRSF13C), myeloid cell activation (PREX1, SBNO2, STXBP2, TGFB1, TICAM1), and cell proliferation (APOA1, ARHGEF1, BRD4, CHRM4, CITED2, E4F1, EMP1, ERF, IRS2, JUNB, KLF10, MMP14, MYC, NF2, and TGFB1). CLL cells heavily depend on interactions with their microenvironment which consists of T cells, dendritic cells, myeloid support cells (NLCs) and myeloid suppressor cells which support the growth and survival of CLL cells. The presence of myeloid derived suppressor cells is correlated with poor clinical outcomes and disease progression [51, 52]. Hence, the expression levels of genes in M10 could potentially serve as markers for CLL progression.

The M2 module has genes involved in bone mineralization (ACVR2B, BMP6, BMPR1A, P2RX7, TGFB3) and cell adhesion (CDH26, FAT2, KIAA0319, PCDH12, PCDHGA1, PCDHGA10, PCDHGA11, PCDHGA12, PCDHGA3, PCDHGB7, and PCDHGC3). Bone loss is a regular occurrence in several types of cancer, including CLL [53]. It is attributed to the increased production of osteoclasts by CLL cells and the effects of chemotherapy on dividing cells [53, 54]. Perhaps the genes in M2 are down-regulated due to CLL cells remodeling their bone marrow microenvironment.

Although M1 did not associate with relapse, this module is of interest due to its association with biomarkers of CLL (i.e., BTK, TP53, BCL2). BCR-signaling is known to activate PI3K/AKT, MEK/ERK, mTOR, and NF-KB signaling to promote cell survival, migration, and proliferation in CLL. The genes in the M1 network are consistent with previous findings and provides novel interactions and functions that are associated with BCR, TP53, and BCL2 signaling. This network is overrepresented with genes involved in glutathione and carbohydrate metabolism. Enhanced glutathione production by CLL cells has been implicated in ROS generating drug resistance and increased expression of anti-apoptotic proteins, such as MCL1 [55] Previously, it has been shown that BTK directly modulates TP53. Normally, BTK activation is increased in response to DNA damage, which ultimately phosphorylates TP53 [56]. However, based on previous findings and the M1 network, CLL cells are protected from ROS induced DNA damage and apoptosis through upregulation of glutathione, oxidoreduction, and anti-apoptotic signals. The M1 co-expression network is the first to implicate connected signaling mechanisms of BCR-signaling and glutathione metabolism. This network also suggests novel signaling mechanisms associated with CLL drug resistance.

Not only has our study shown the potential value of using gene expression modules to characterize CLL biology, but we have also shown that they can be used to stratify patients into biological sub-groups. However, our clinical conclusions on module-defined patient sub-groups are limited due to the amount of clinical information available for the ICGC cohort. We identified seven patient sub-groups after hierarchal clustering of module eigengene expression (Fig. 4B). 3 groups (clusters 2, 4, and 5) represented distinct relapse risk groups. Previously, CLL was stratified into two groups (C1 and C2) based on gene expression data [57] In our study, we observed that ICGC clusters 2 and 4 (increased relapse risk groups) have above average expression of genes in the M3 and M4 modules and below average expression of M1 and M2 whereas Cluster 5 had the opposite expression patterns and a lower relapse risk. In the aforementioned study, group C2 had lower time to treatment intervals and increased expression of RNA splicing, mRNA transport, MAPK signaling, and organic substance response genes. Similarly, we observed the overexpression of modules with genes involved in mRNA and macromolecule signaling pathways in relapsed patients, which are the majority of the patients in ICGC clusters 2 and 4. Perhaps patients in module defined sub-groups (clusters 2 and 4) have similar biology to the C2 group.

In this study, we propose CRY1, URAHP, MID1IP1, and CLEC3B as biomarkers for relapse risk in CLL. CRY1 has been implicated as a prognostic marker progression and survival in CLL and other types of cancer, however, the role of URAHP, MID1IP1, and CLEC3B has not been explored in CLL [58, 59]. We also propose ARHGAP27P2, CASC2, CXCR5, C1S, and FUT5 as markers to predict overall survival and relapse free survival. FUT5 and C1S have been implicated as biomarkers for differentiation, proliferation, migration, and invasion in other cancers [60–62]. High CASC2 expression has been previously shown to inhibit pancreatic and ovarian cancer growth and, opposingly, contribute to breast cancer chemoresistance [63–65]. CXCL13-CXCR5 signaling is currently a biomarker for metastasis in breast and prostate cancer and mortality in colorectal cancer [66–68]. Our study is the first to report CASC2, CXCR5, and ARHGAP27P2 as biomarkers for poor overall survival prognosis in CLL. The roles of these novel biomarker genes in CLL should be further studied in silico and in vitro.

## Conclusion

This is the first study to use WGCNA to identify gene signatures in CLL. Mechanisms responsible for relapse have not been completely characterized. Our study identified eight networks associated with relapse and three associated with overall survival. The modules represent known and novel pathways associated with CLL pathogenesis and relapse and can be a resource for the CLL research community. The hub genes of these modules, e.g., ARHGAP27P2, C1S, CASC2, CLEC3B, CRY1, CXCR5, FUT5, MID1IP1, and URAHP, can be studied further as new therapeutic targets or can be used as clinical markers to predict patient outcomes.

## Supplementary Information

**Additional file 1.** Module Preservation of ICGC CLL Modules in the Broad CLL Dataset. Median rank (left figure, y-axis) of modules determined by module preservation scores (right, y-axis) are displayed. Modules ranked closest to zero are the most preserved. The blue and red dotted lines denote cutoffs for a –log rank *p* value of 0.05 (blue) and *p* value < 0.00001(red). Most modules (10 out of 13) are preserved in the Broad CLL dataset.

**Additional file 2.** Confounding Variable Variance. Variance scores (x-axis) of confounding variables (bold) and the top 25 genes (**A**, y-axis) and modules (**B**, y-axis) affected. At the individual gene level, age and sex contribute to less than 10% to the variance in gene expression of the top affected genes. At the module level, age at diagnosis and IGHV status are the largest contributors to variance in gene expression. 8 of 13 modules are minimally affected by confounding variables.

**Additional file 3.** Module eigengenes and their gene membership. Genes are listed, followed by their membership scores for each module, and lastly the module that they were assigned to.

**Additional file 4.** Module-trait Relationships of regressed ICGC data based on IGHV Status. Modules for Unmutated IGHV (**A**) and Mutated IGHV (**B**) datasets are denoted on the x-axis, traits on y-axis. The blue-white-red colors inside of the heatmap indicate positive (red), negative (blue), and no (white) correlations. The numbers, inside the heatmaps, represent the correlation test *p* values. WGCNA of unmutated patient data produced more modules (17 vs 11) than the mutated dataset.

**Additional file 5.** Module Preservation of Modules based on Regression and IGHV Status. WGCNA’s Module Preservation analysis was applied according to regression (**A**, **B**) and IGHV (**C**, **D**) status. Median rank (left figure, y-axis) of modules determined by module preservation scores (right figure, y-axis) are displayed. Modules ranked closest to zero are the most preserved. The blue and red dotted lines denote cutoffs for a –log rank *p* value of 0.05 (blue) and *p* value < 0.00001. Module gene correlations are preserved regardless of IGHV and Regression status.

**Additional file 6.** Module-Trait Heatmaps of Regressed and Unregressed ICGC Data (.pdf). Modules for unregressed (**A**) and regressed (**B**, **C**) datasets are denoted on the x-axis, traits on y-axis. **B** Modules-trait relationships following regression of sex and age. **C** reprEsents modules-trait relationships following regression of sex, age, and IGHV status. The blue-white-red colors inside of the heatmap indicate positive (red), negative (blue), and no(white) correlations. The numbers, inside the heatmaps, represent the correlation test *p* values. WGCNA of unregressed data produced more modules (13 vs 11) and significant module-trait relationships (45 vs 34) than the regressed (panel B) dataset.

**Additional file 7.** Modules with altered expression in Unmutated CLL patients based on SF3B1 and Relapse Status. Violin plots of module eigengene (red and brown) expression (x-axis) based on SF3B1 (**A**) and relapse status (**B**). A Wilcoxon test was used to determine if module expression was altered between groups.

**Additional file 8.** Modules with altered expression in Mutated CLL patients based on SF3B1 and Relapse Status. Violin plots of module eigengene tan, red, greenyellow, purple, yellow) expression (x-axis) based on SF3B1 (**A**) and relapse status (**B**). A Wilcoxon test was used to determine if module expression was altered between groups.

**Additional file 9.** Gene Ontology of Relapse-Associated M-CLL network modules. Biological processes (green), molecular processes (blue), and cellular locations (brown) of M6, M10, M11, and M12 modules are displayed on the y-axis. Z-scores are on the x-axis.

**Additional file 10.** Gene Ontology of Relapse (U-M3) and SF3B1(U-M6) mutation associated U-CLL modules. Biological processes (green), molecular processes (blue), and cellular locations (brown) of are displayed on the y-axis. Z-scores are on the x-axis.

**Additional file 11.** ANOVA Differential Expression Results. The genes are listed, followed by ANOVA F statistics, Tukey *p* values, log2 FPKM differences, and module names.

**Additional file 12.** Optimum RFS Gene Combination AUC in All ICGC CLL patients. Receiver Operator curves for Optimum biomarker gene combinations are shown for each time point: 15 months (**A**), 18 months (**B**), 3 years (**C**), and 5 years (**D**), The y-axis represents the percentage of patients who were true positives for relapse, whereas the x-axis represents the percentage of patients who were true negatives. The AUC (top left legend) for each time point is represented by a distinct color: dark orange (15 months), red (18 months), dark red (3 years), and magenta (5 years).

**Additional file 13.** Optimum RFS Gene Combination AUC in U-CLL patients. Receiver Operator curves for Optimum biomarker gene combinations are shown for each time point: 15 months (**A**), 18 months (**B**), 3 years (**C**), and 5 years (**D**), The y-axis represents the percentage of patients who were true positives for relapse, whereas the x-axis represents the percentage of patients who were true negatives. The AUC (top left legend) for each time point is represented by a distinct color: dark orange (15 months), red (18 months), dark red (3 years), and magenta (5 years).

**Additional file 14.** AUC Gene Combinations Tested in All ICGC CLL patients. Biomarker Gene combinations are listed, followed by their AUC scores for each time point (15 months, 18 months, 3 years, and 5 years),

**Additional file 15.** AUC Gene Combinations Tested in U-CLL ICGC CLL patients. Biomarker Gene combinations are listed, followed by their AUC scores for each time point (15 months, 18 months, 3 years, and 5 years).

**Additional file 16.** Overall Survival of hub genes from networks associated with survival days. OS for nine hub genes from the M3, M7, and M13 networks were evaluated. The red lines denote OS of patients with high gene expression and the blue lines refer to patients with low expression.

**Additional file 17.** Relapse Free Survival of hub genes from Relapse associated networks. RFS for twelve hub genes from the M3, M7, M10, and M13 networks were evaluated. The red lines denote RFS of patients with high gene expression and the blue lines refer to patients with low expression.

## Data Availability

The RNA sequencing datasets analyzed during the current study are the International Cancer Genome Consortium CLLE-ES project dataset (https://dcc.icgc.org/projects/CLLE-ES) and cBioPortal Broad CLL dataset (http://www.cbioportal.org/study/summary?id=cll_broad_2015).

## Abbreviations

BCR: B cell receptor
CLL: Chronic lymphocytic leukemia
CS: Confidence scores
GIANT: Genome-wide Integrated Analysis of gene Networks in Tissues
GO: Gene ontology
GSEA: Gene set enrichment analysis
FCR: Fludarabine, cyclophosphamide, rituximab
ICGC: International Cancer Genome Consortium
M-CLL: Mutated CLL
ME: Module eigengene
OS: Overall survival
RFS: Relapse free survival
ROC: Receiver operator curve
RPKM: Reads per kilobase per million
U-CLL: Unmutated CLL
WGCNA: Weighted Gene Co-expression Network Analysis
Z.K.: Z-transformed sample connectivity
