# Supplementary figures and images for: Multivariate transcriptome analysis identifies networks and key drivers of chronic lymphocytic leukemia relapse risk and patient survival
Source: BMC Med Genomics. 2021 Jun 29;14:171. doi: 10.1186/s12920-021-01012-y (PMC8243588; doi:10.1186/s12920-021-01012-y)

medianRank Preservation

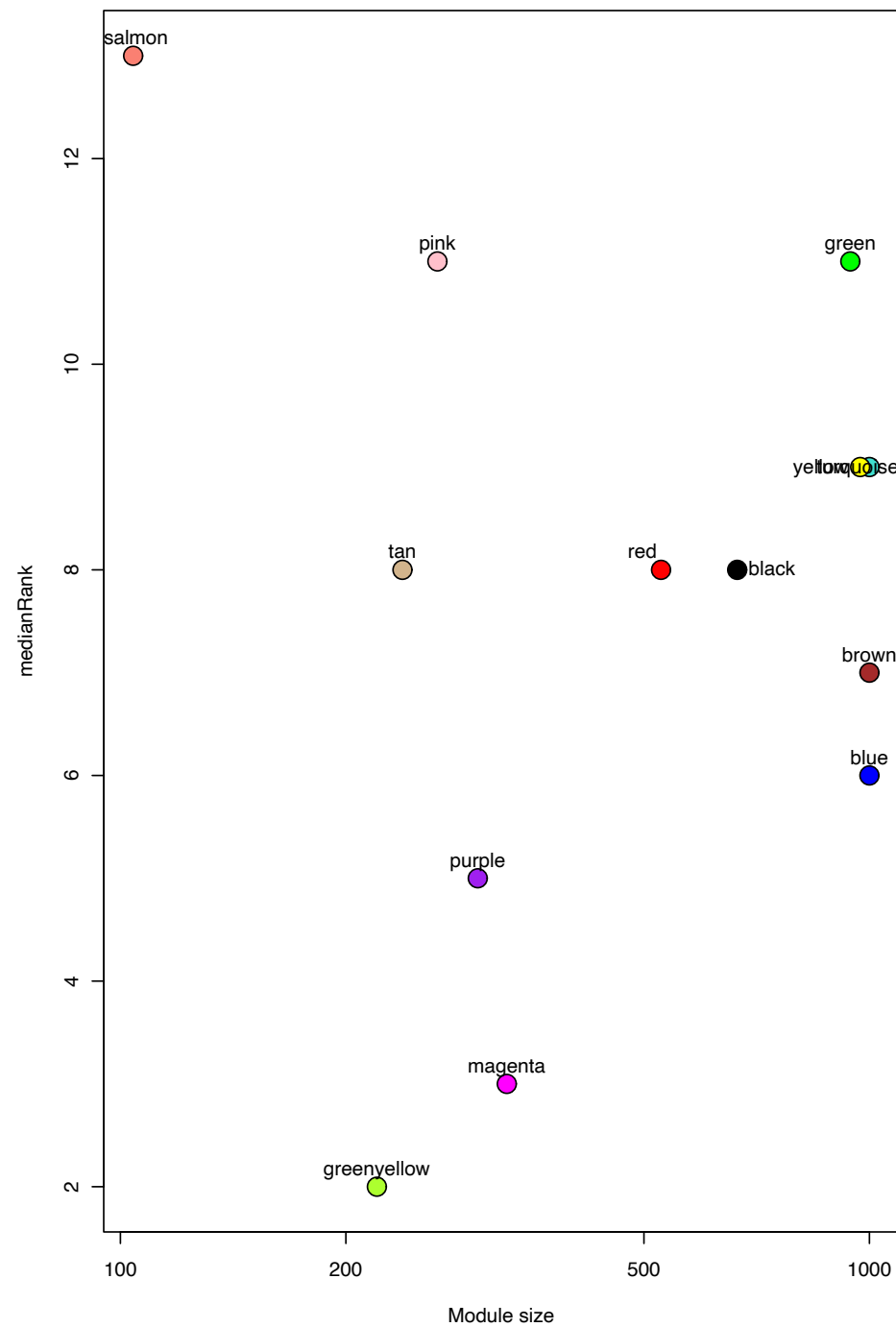

Zsummary Preservation

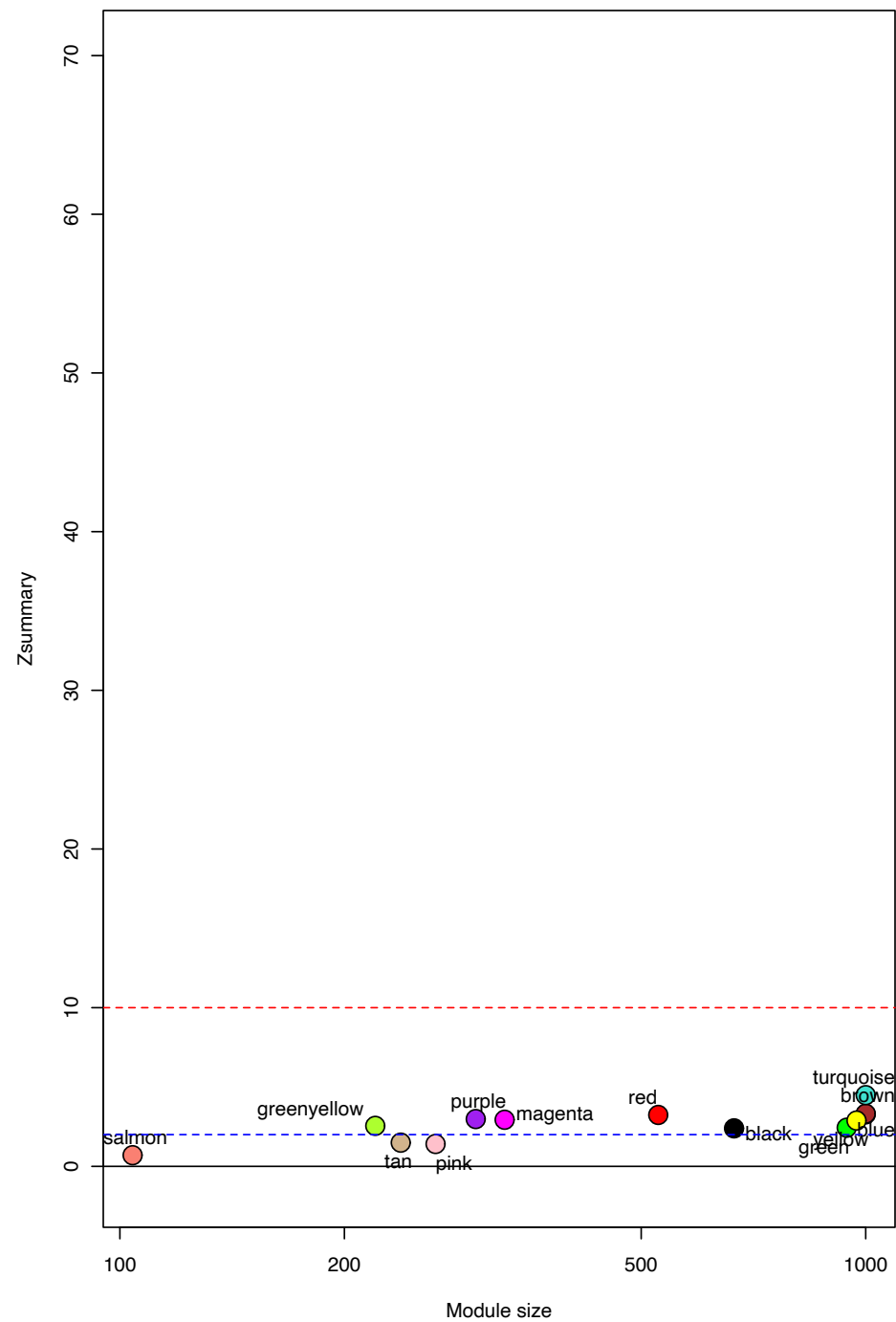

Supplement: Supplementary file 1 — Additional file 1. Module Preservation of ICGC CLL Modules in the Broad CLL Dataset. Median rank (left figure, y-axis) of modules determined by module preservation scores (right, y-axis) are displayed. Modules ranked closest to zero are the most preserved. The blue and red dotted lines denote cutoffs for a –log rank p value of 0.05 (blue) and p value < 0.00001(red). Most modules (10 out of 13) are preserved in the Broad CLL dataset. [file 12920_2021_1012_MOESM1_ESM.pdf]

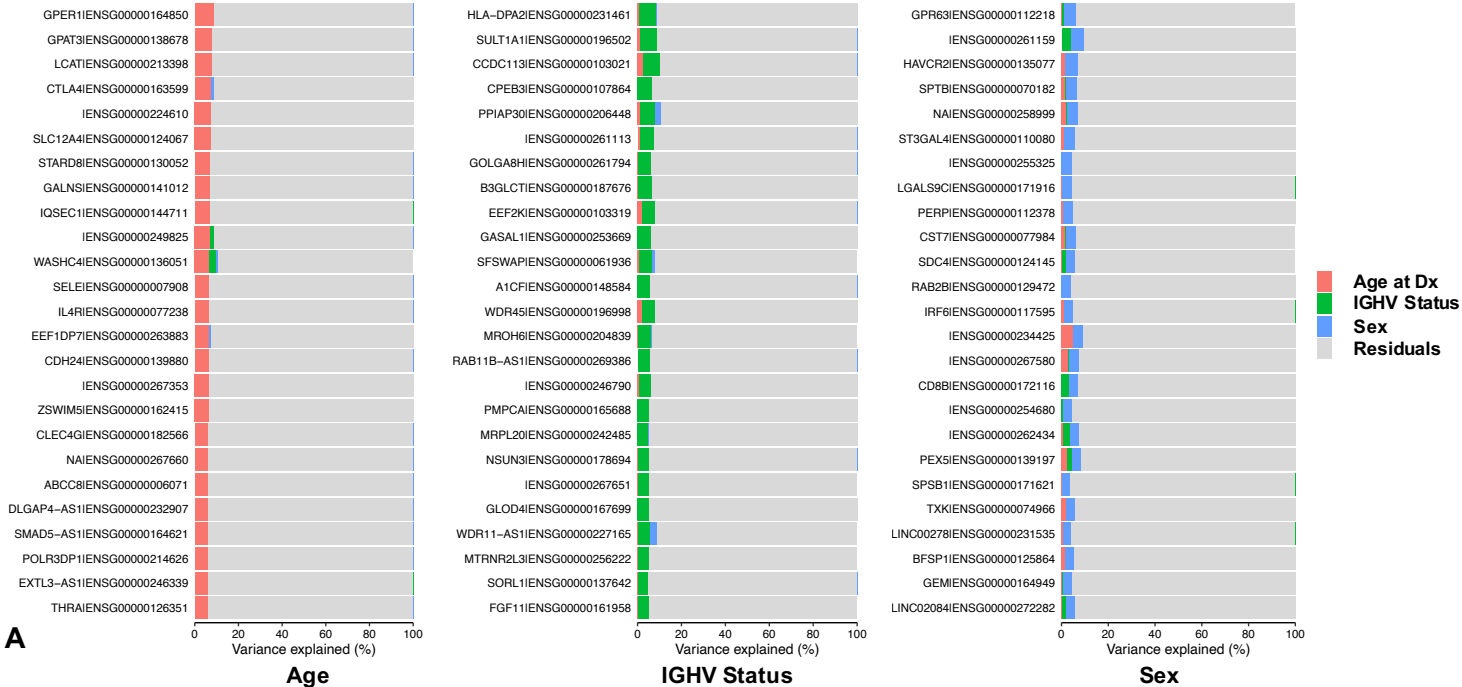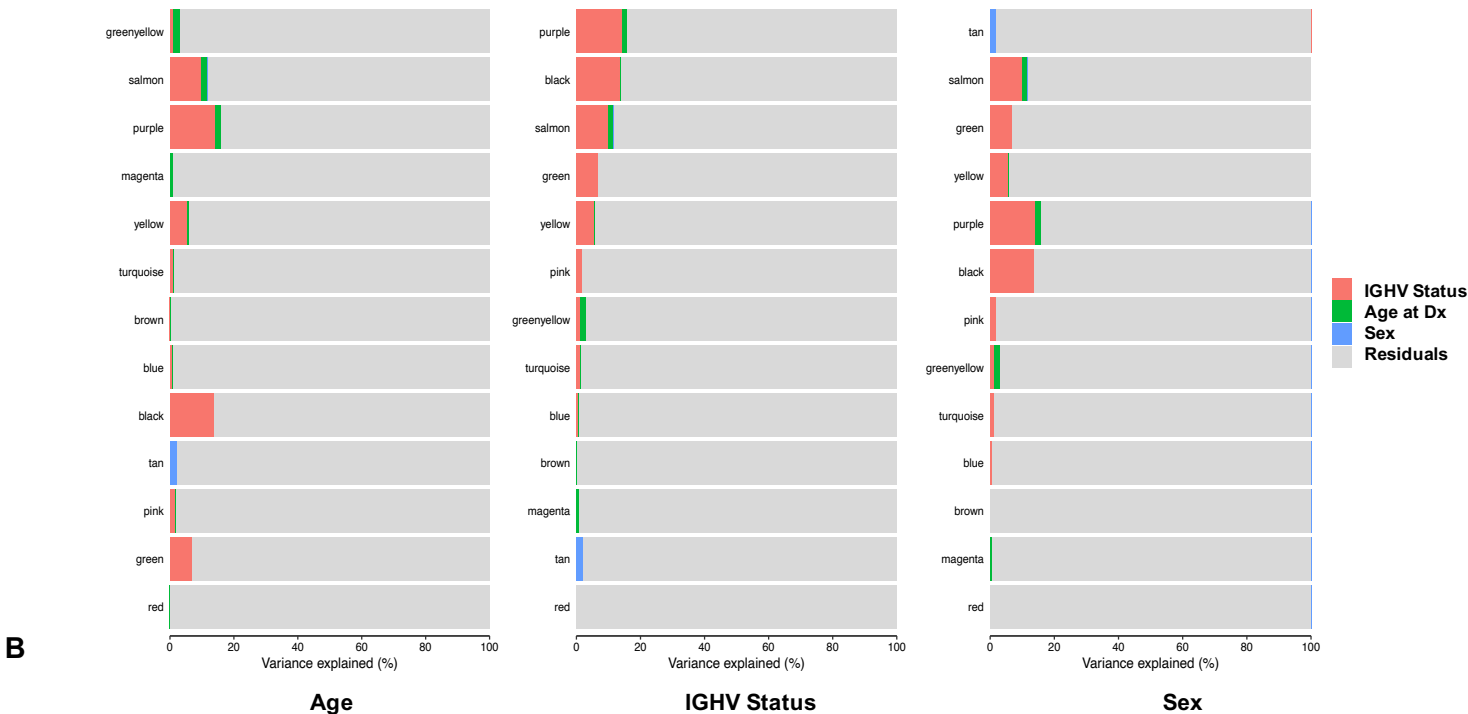

Supplement: Supplementary file 2 — Additional file 2. Confounding Variable Variance. Variance scores (x-axis) of confounding variables (bold) and the top 25 genes (A, y-axis) and modules (B, y-axis) affected. At the individual gene level, age and sex contribute to less than 10% to the variance in gene expression of the top affected genes. At the module level, age at diagnosis and IGHV status are the largest contributors to variance in gene expression. 8 of 13 modules are minimally affected by confounding variables. [file 12920_2021_1012_MOESM2_ESM.pdf]

# A

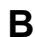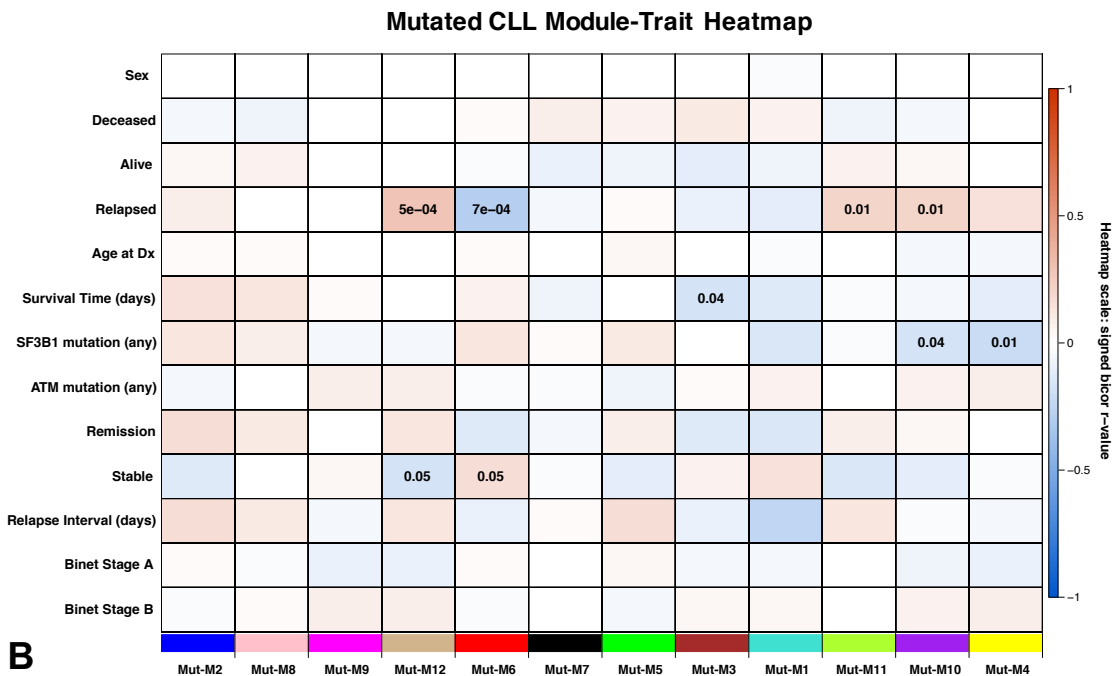

Supplement: Supplementary file 4 — Additional file 4. Module-trait Relationships of regressed ICGC data based on IGHV Status. Modules for Unmutated IGHV (A) and Mutated IGHV (B) datasets are denoted on the x-axis, traits on y-axis. The blue-white-red colors inside of the heatmap indicate positive (red), negative (blue), and no (white) correlations. The numbers, inside the heatmaps, represent the correlation test p values. WGCNA of unmutated patient data produced more modules (17 vs 11) than the mutated dataset. [file 12920_2021_1012_MOESM4_ESM.pdf]

SF3B1mut Wildtype Mutated

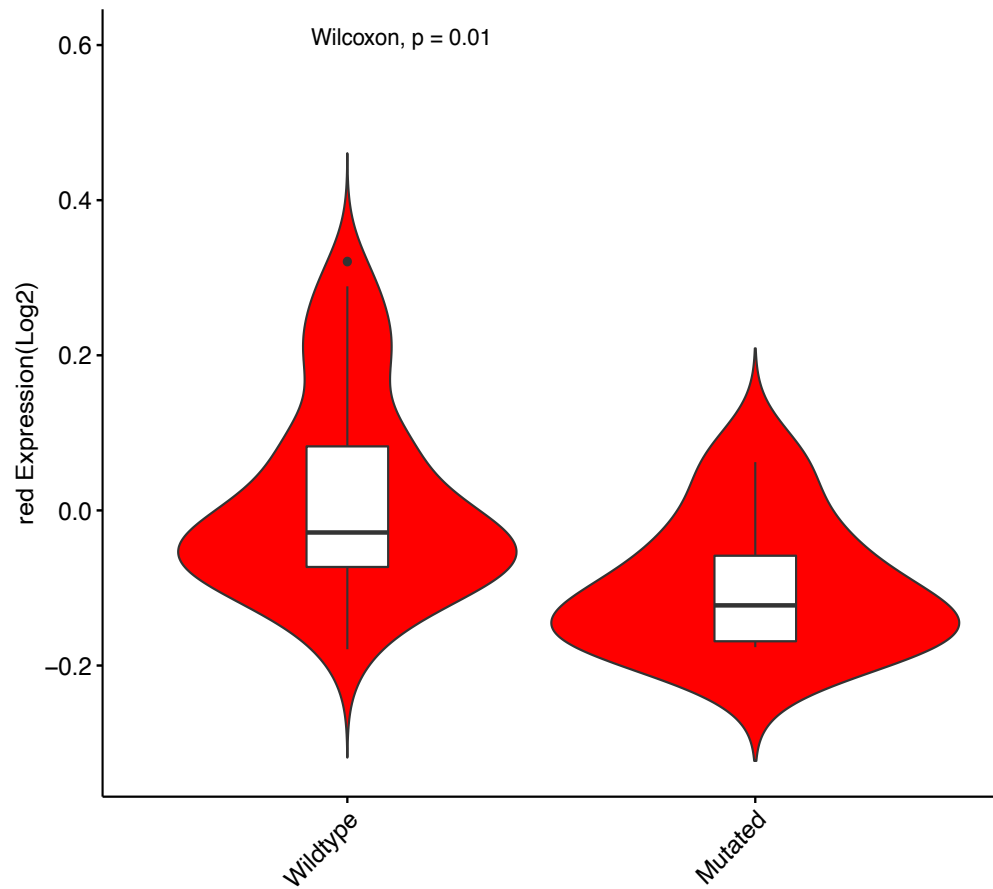

**A**

BinetStageARelapse StageANoRelapse StageARelapsed

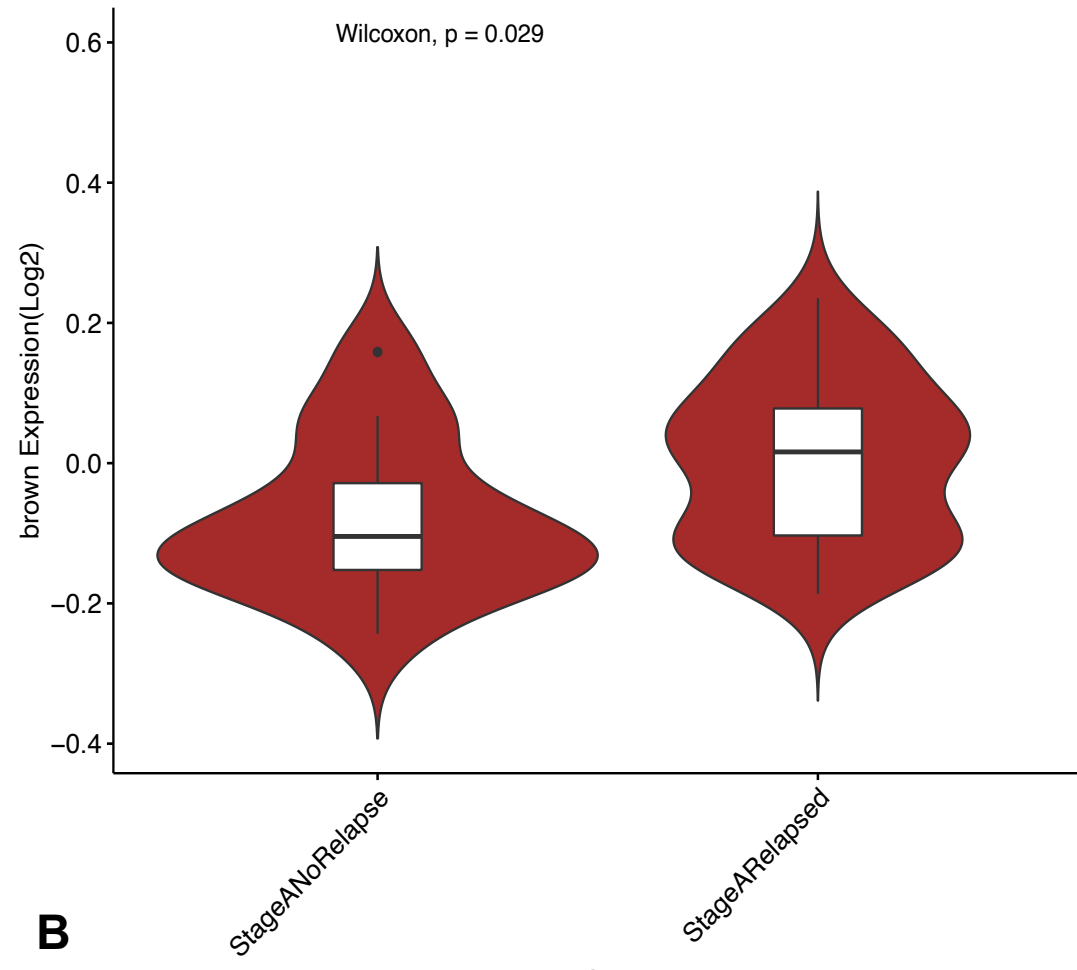

**B**

Supplement: Supplementary file 7 — Additional file 7. Modules with altered expression in Unmutated CLL patients based on SF3B1 and Relapse Status. Violin plots of module eigengene (red and brown) expression (x-axis) based on SF3B1 (A) and relapse status (B). A Wilcoxon test was used to determine if module expression was altered between groups. [file 12920_2021_1012_MOESM7_ESM.pdf]

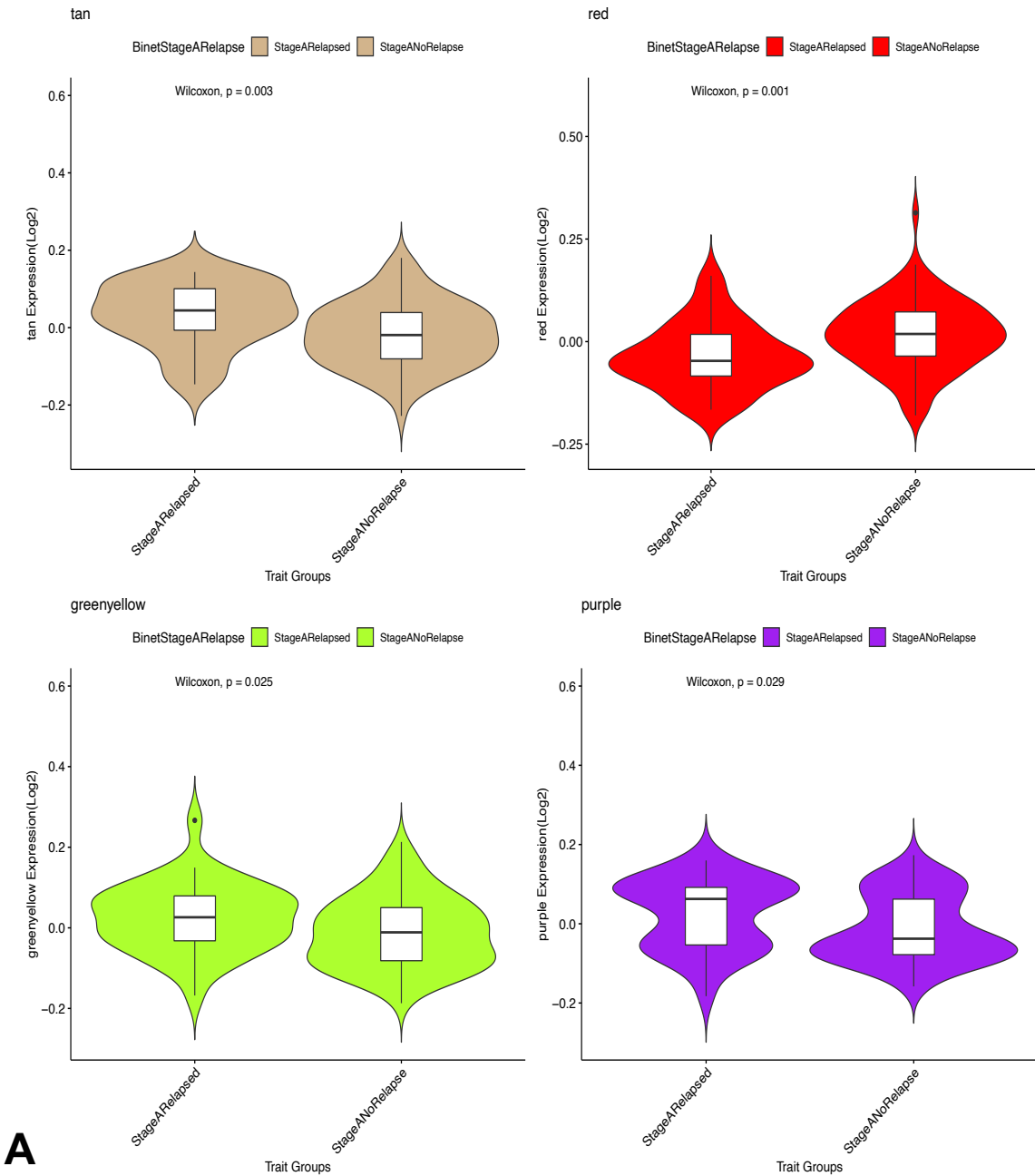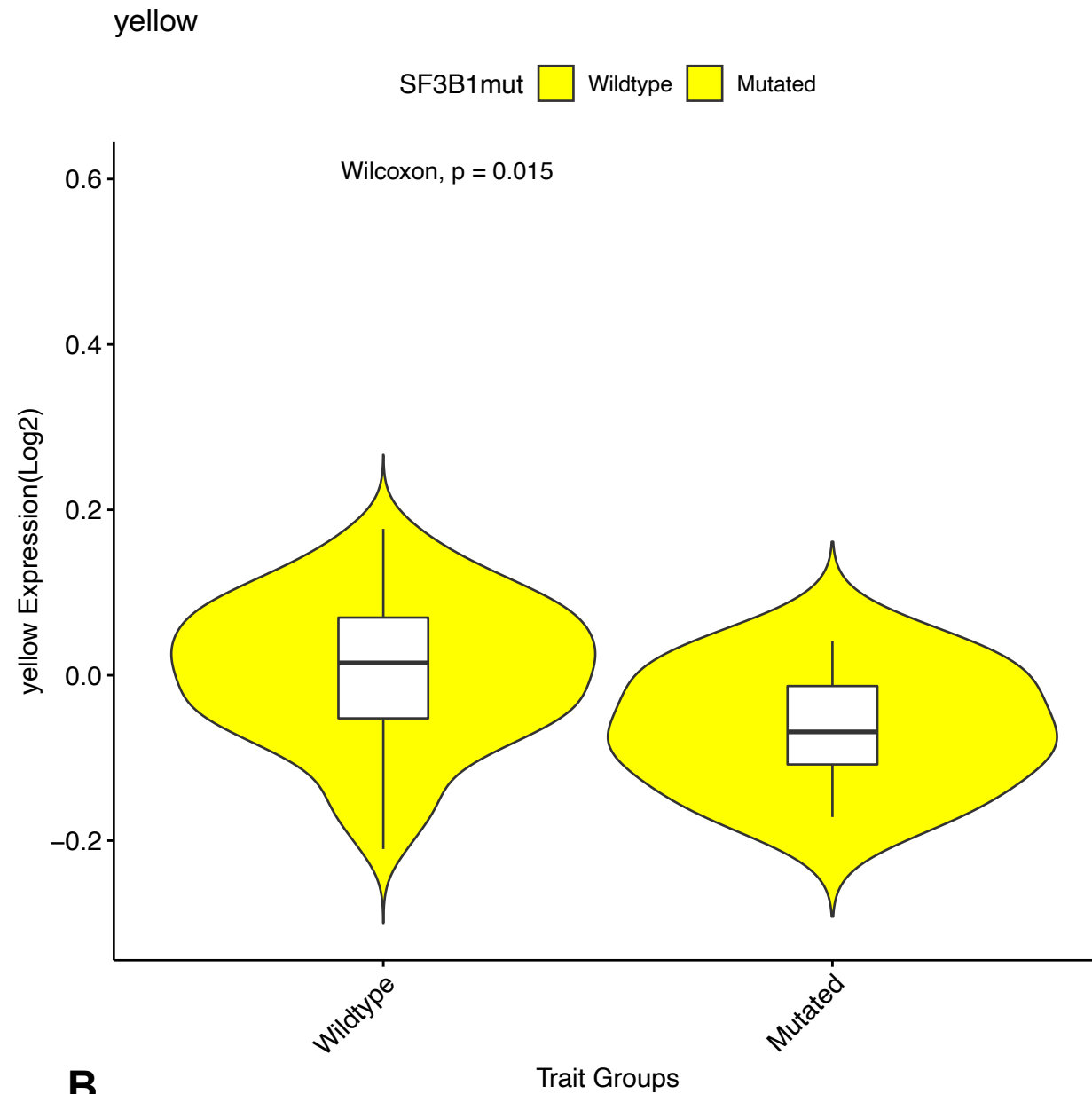

Supplement: Supplementary file 8 — Additional file 8. Modules with altered expression in Mutated CLL patients based on SF3B1 and Relapse Status. Violin plots of module eigengene tan, red, greenyellow, purple, yellow) expression (x-axis) based on SF3B1 (A) and relapse status (B). A Wilcoxon test was used to determine if module expression was altered between groups. [file 12920_2021_1012_MOESM8_ESM.pdf]

M6 red

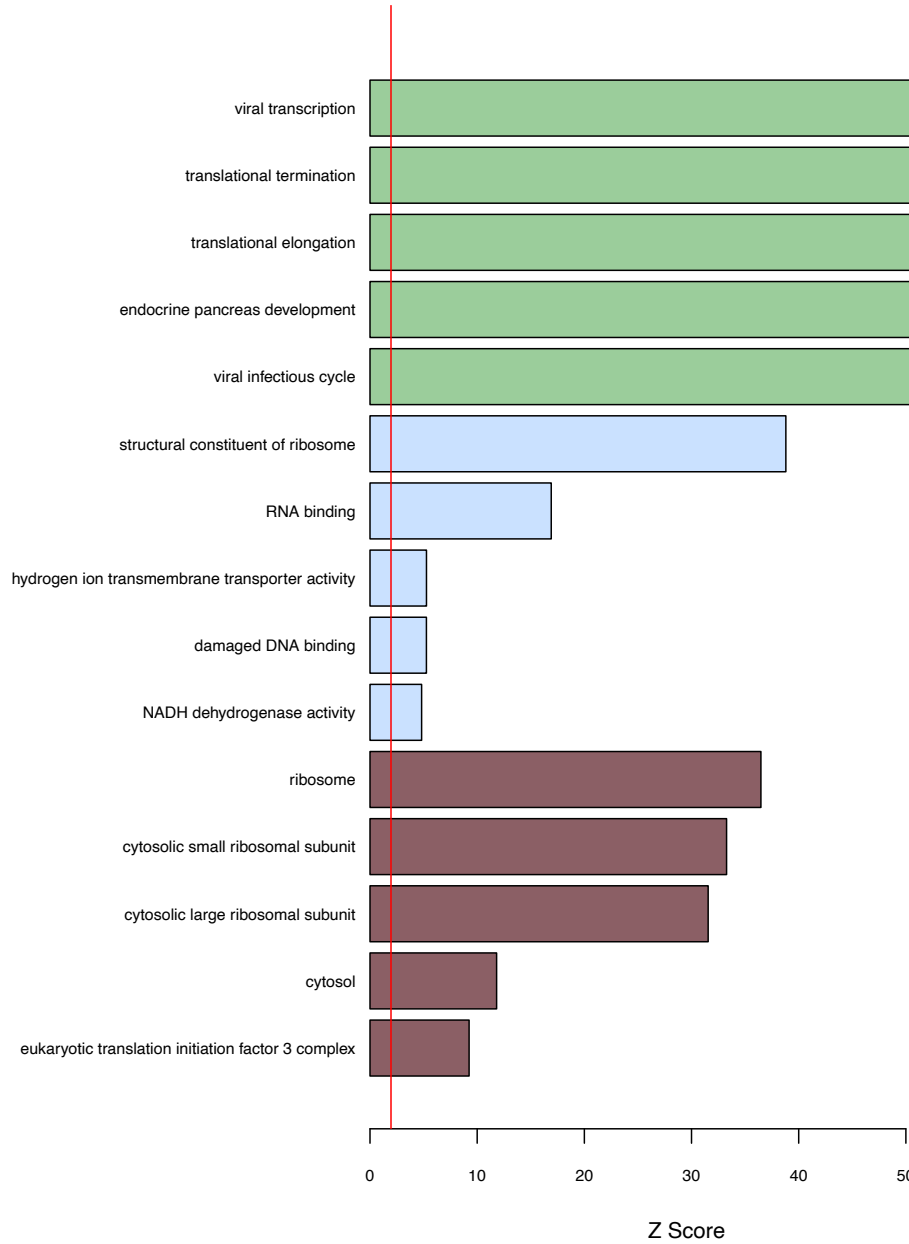

M10 purple

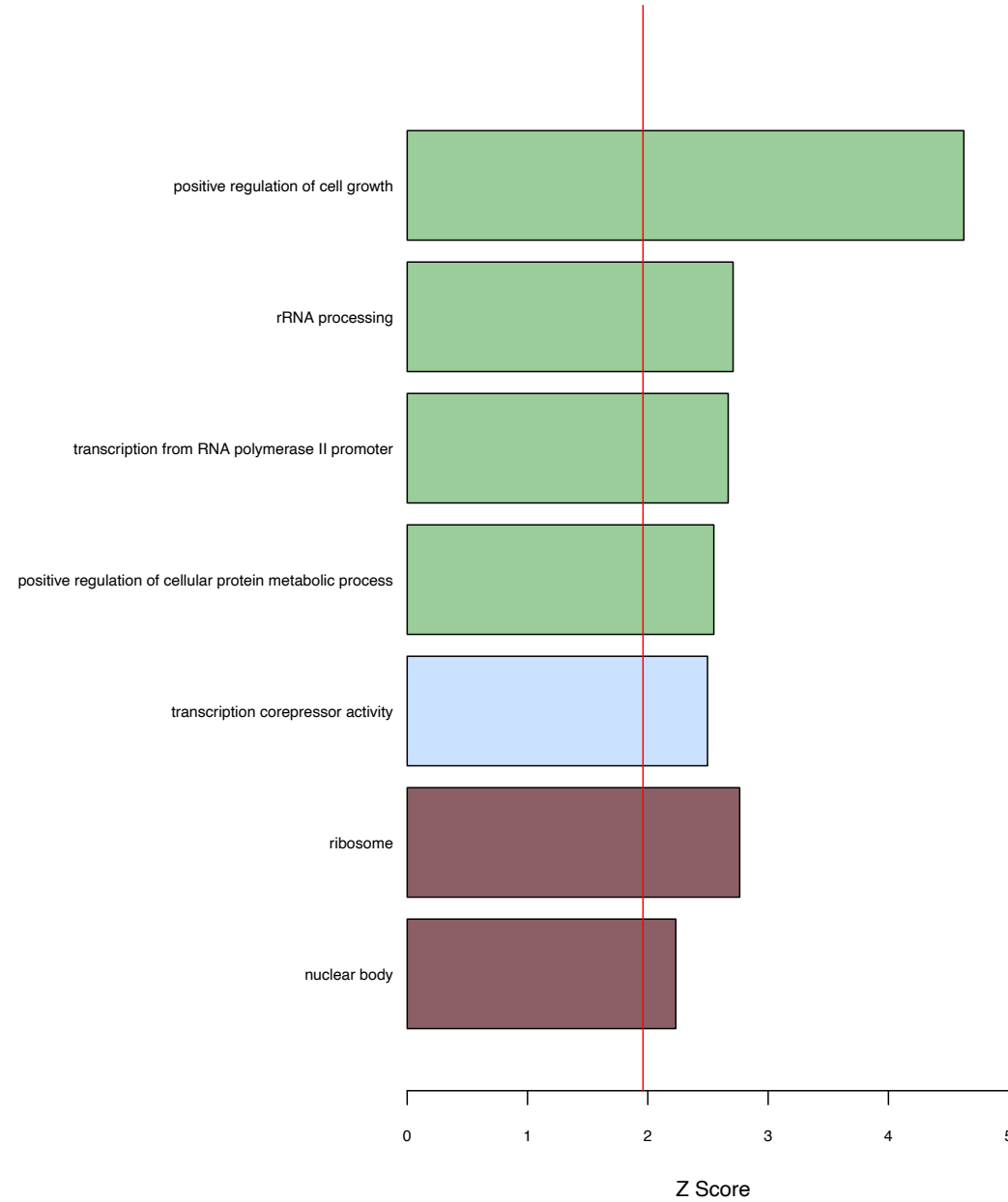

M11 greenyellow

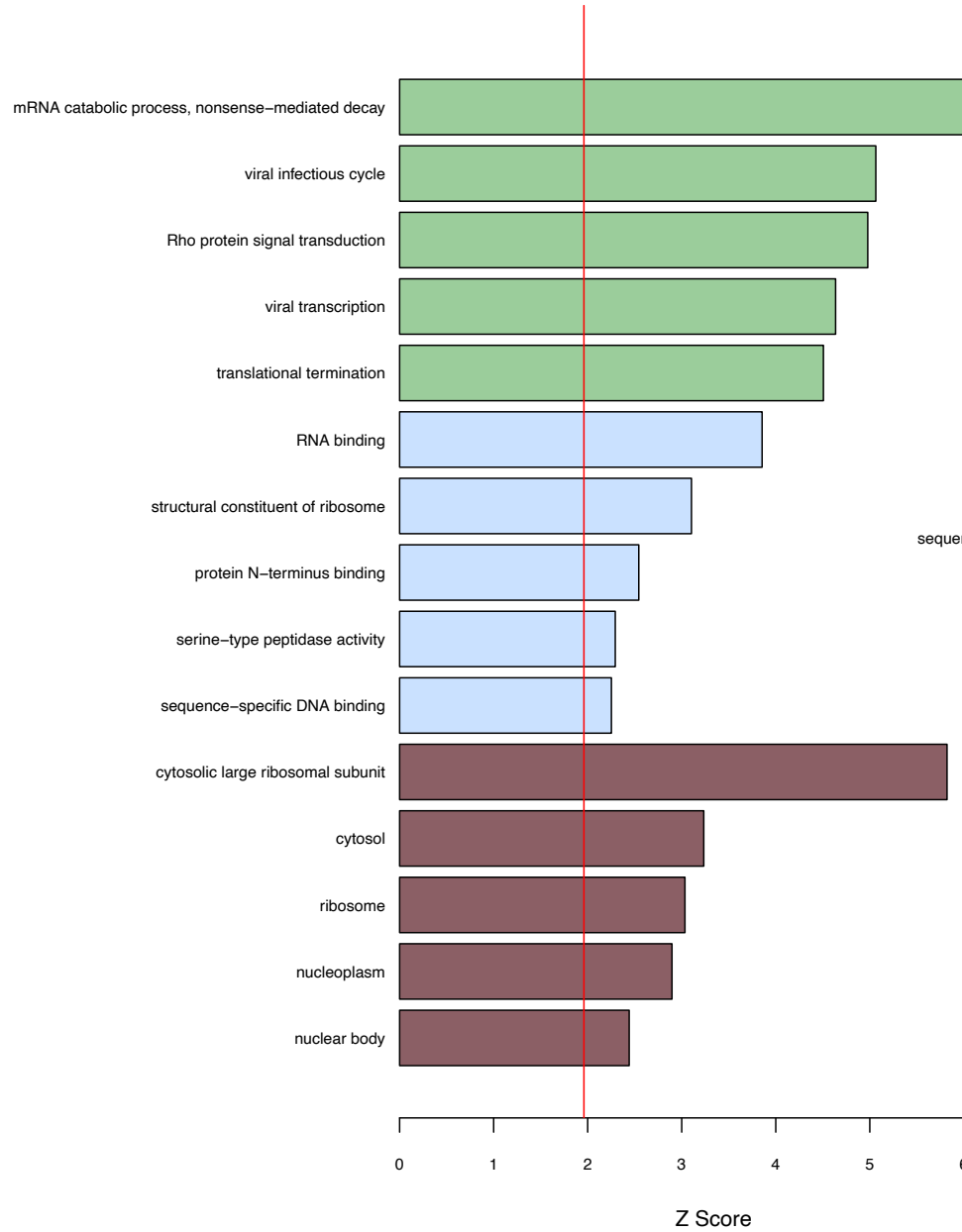

M12 tan

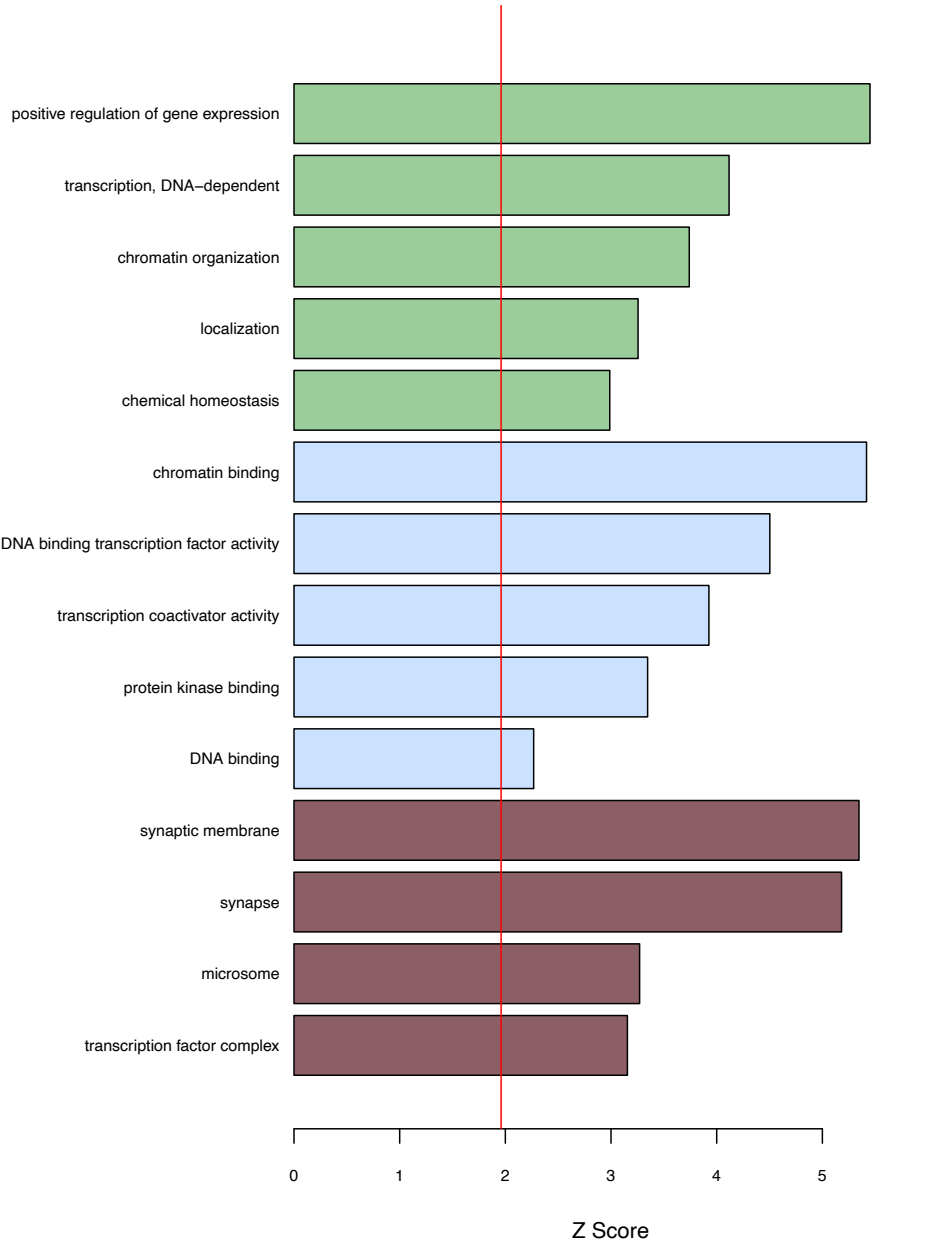

Supplement: Supplementary file 9 — Additional file 9. Gene Ontology of Relapse-Associated M-CLL network modules. Biological processes (green), molecular processes (blue), and cellular locations (brown) of M6, M10, M11, and M12 modules are displayed on the y-axis. Z-scores are on the x-axis. [file 12920_2021_1012_MOESM9_ESM.pdf]

M3 brown

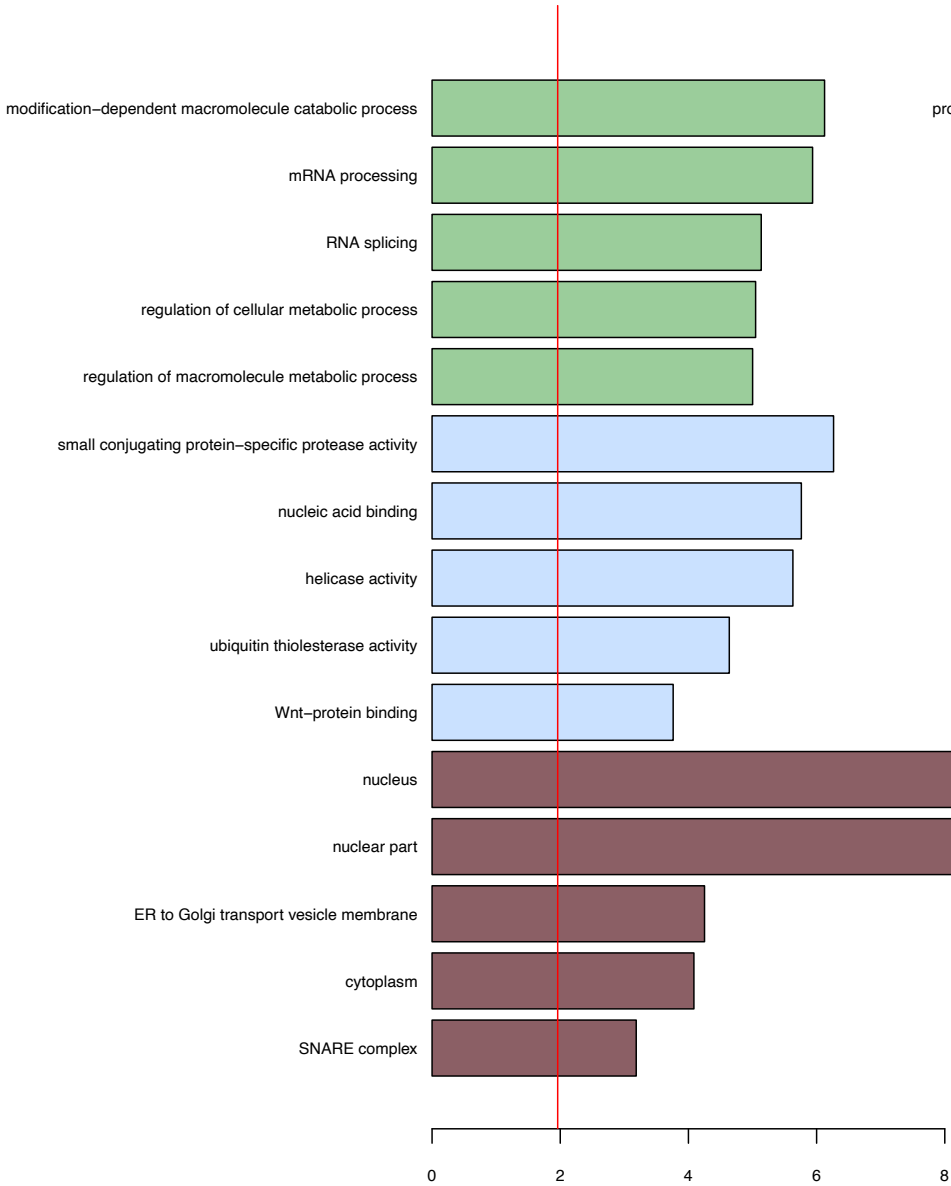

M6 red

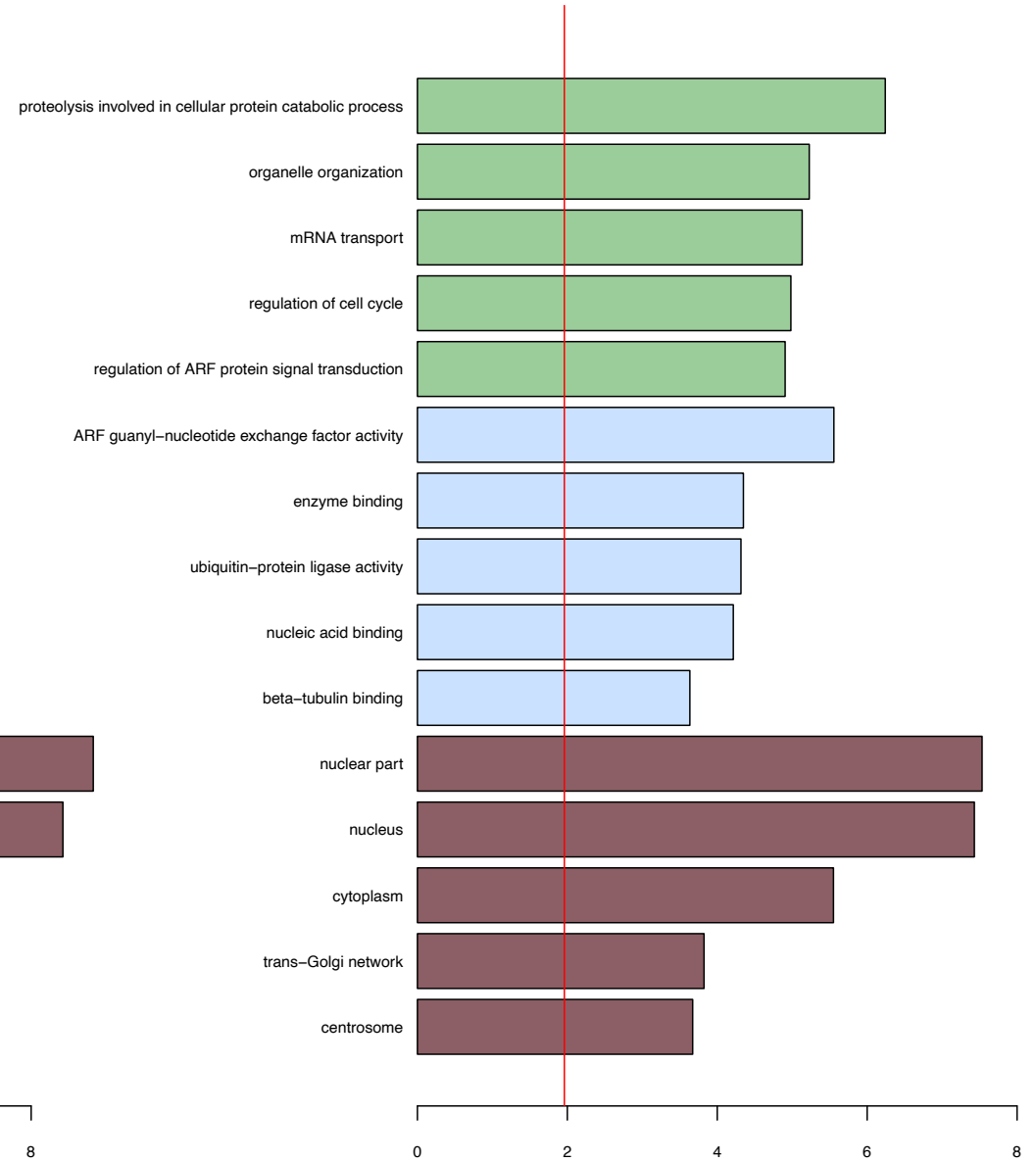

Supplement: Supplementary file 10 — Additional file 10. Gene Ontology of Relapse (U-M3) and SF3B1(U-M6) mutation associated U-CLL modules. Biological processes (green), molecular processes (blue), and cellular locations (brown) of are displayed on the y-axis. Z-scores are on the x-axis. [file 12920_2021_1012_MOESM10_ESM.pdf]

### SLC7A5+URAHP+FAM166A/CNTNAP2+HOMER3+MACC1

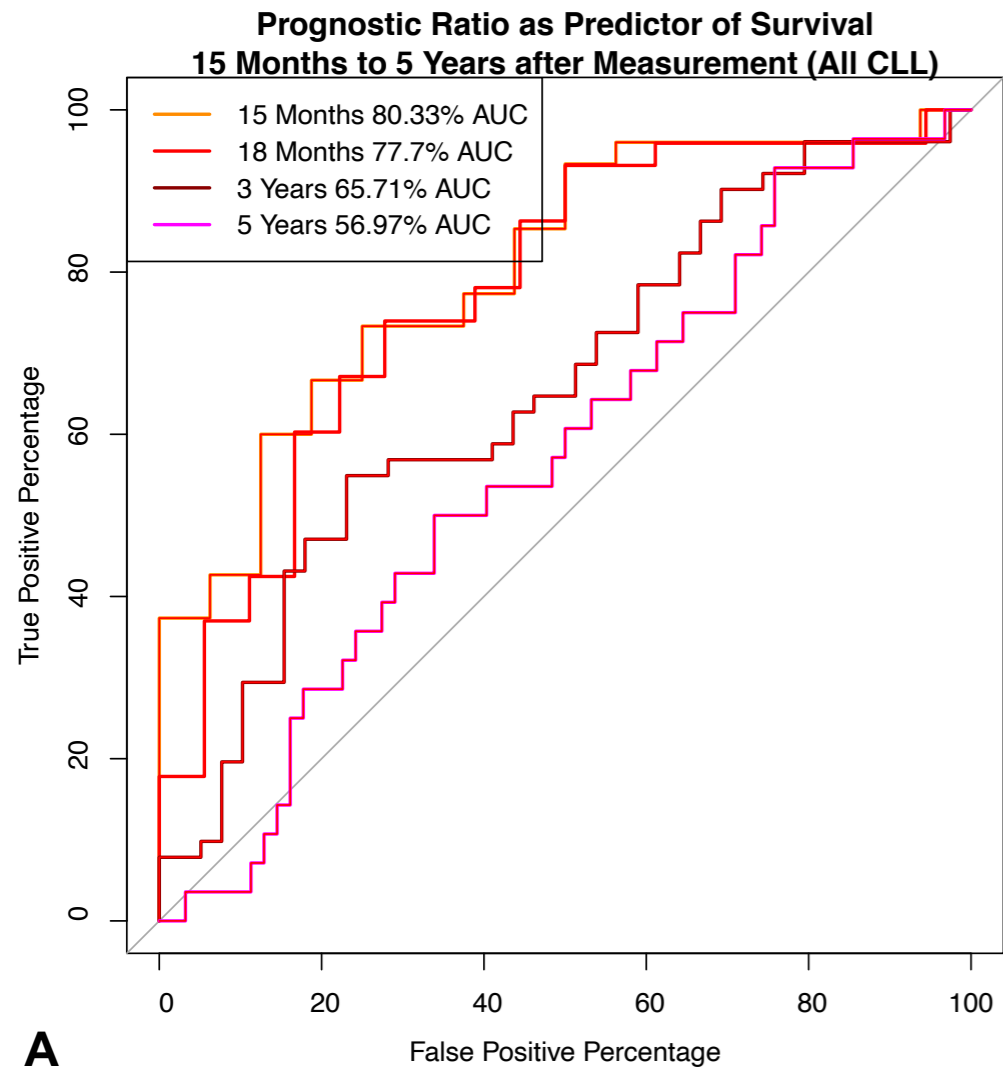

### ID1+LPL+FUT5/SNHG25+APOD+ADAM29

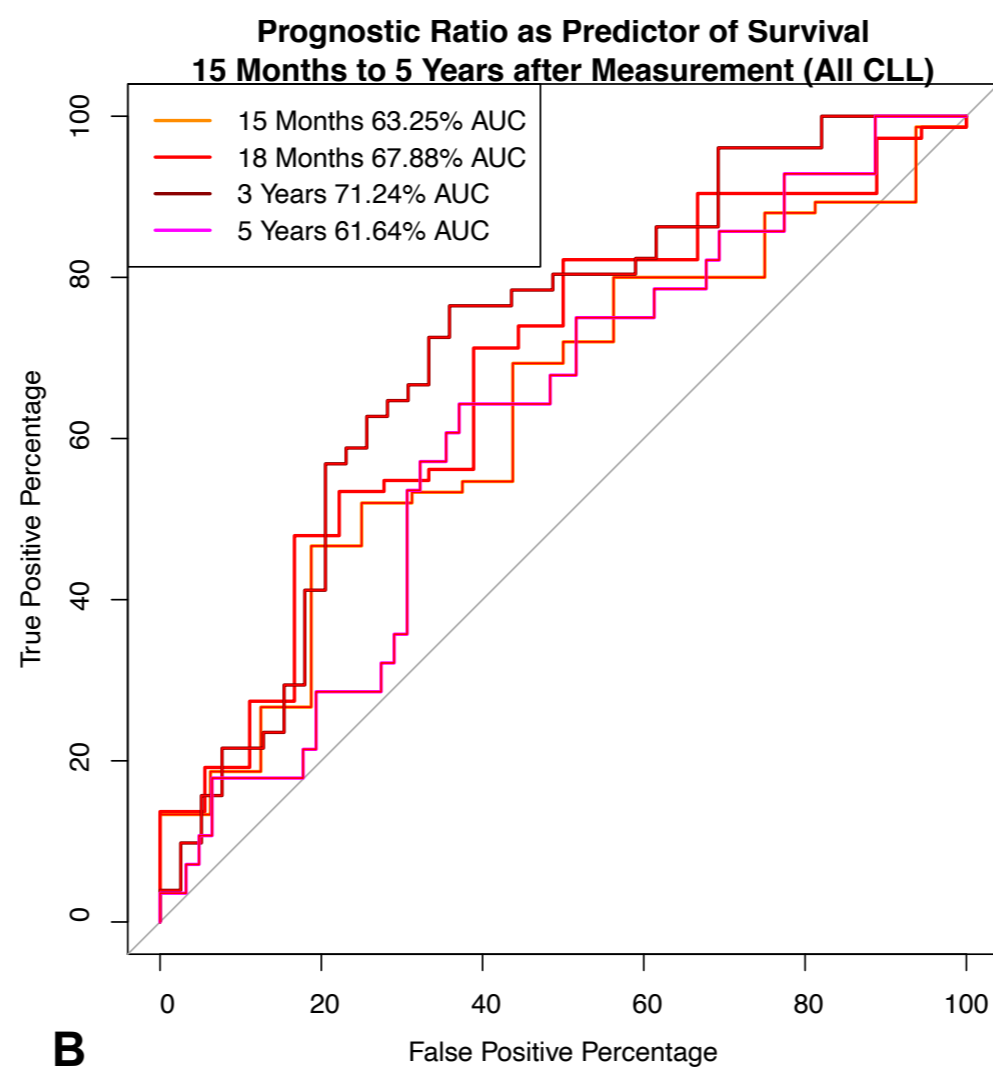

### C1S+TCTEX1D4/CNTNAP2+HOMER3

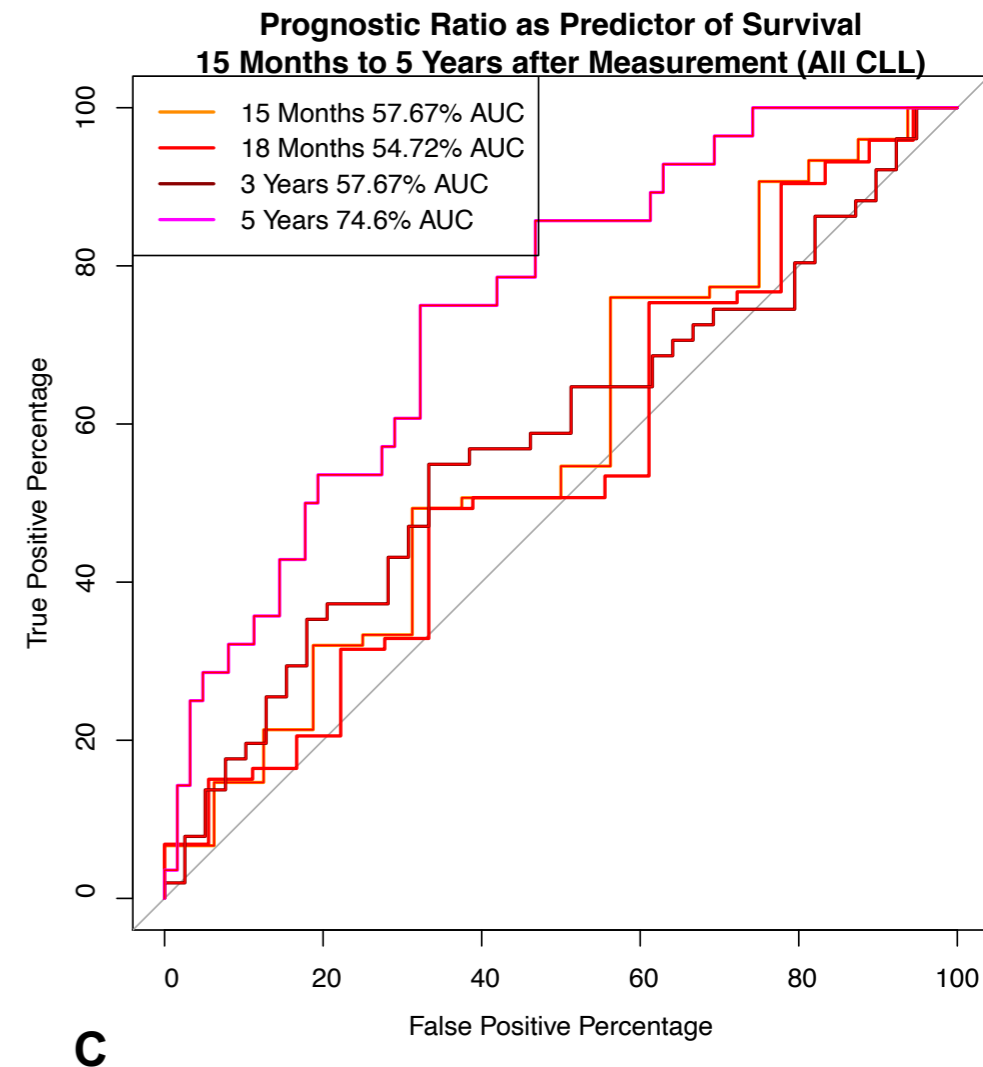

Supplement: Supplementary file 12 — Additional file 12. Optimum RFS Gene Combination AUC in All ICGC CLL patients. Receiver Operator curves for Optimum biomarker gene combinations are shown for each time point: 15 months (A), 18 months (B), 3 years (C), and 5 years (D), The y-axis represents the percentage of patients who were true positives for relapse, whereas the x-axis represents the percentage of patients who were true negatives. The AUC (top left legend) for each time point is represented by a distinct color: dark orange (15 months), red (18 months), dark red (3 years), and magenta (5 years). [file 12920_2021_1012_MOESM12_ESM.pdf]

ARHGAP27P2

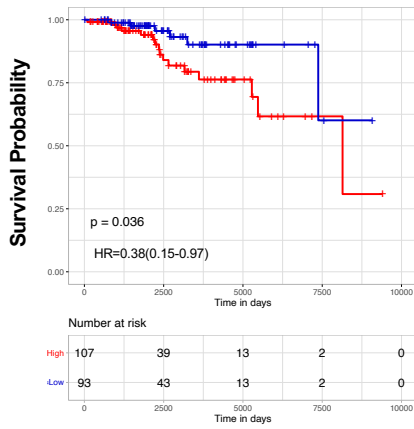

CASC2

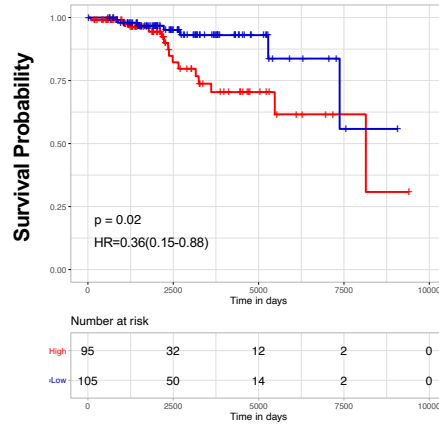

C4ORF48

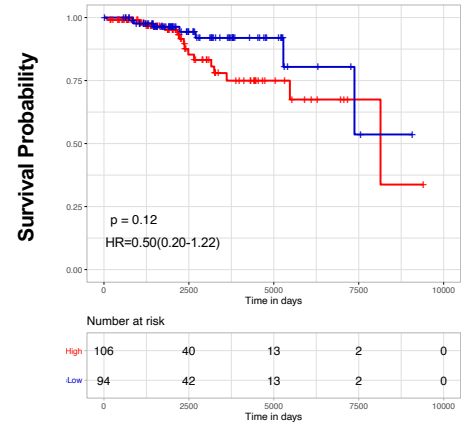

HSPBP1

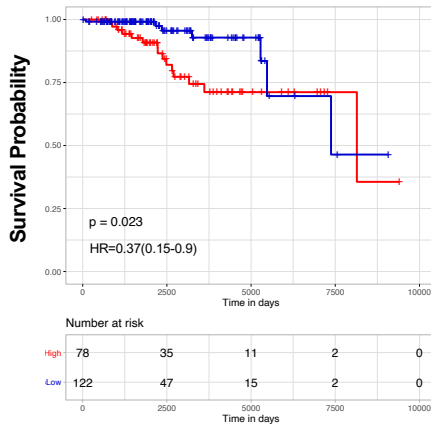

FUT5

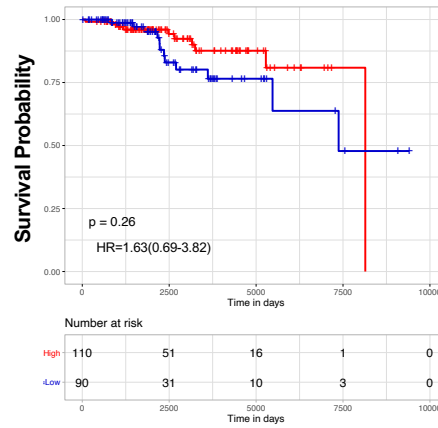

C1S

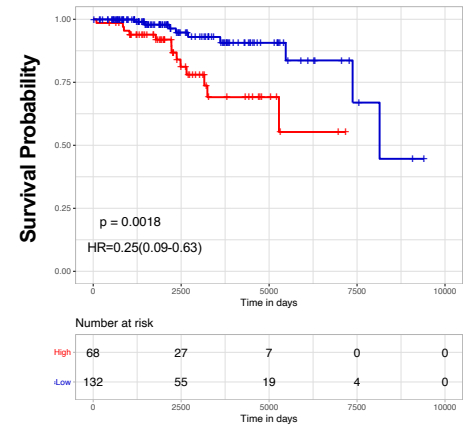

PTPRH1

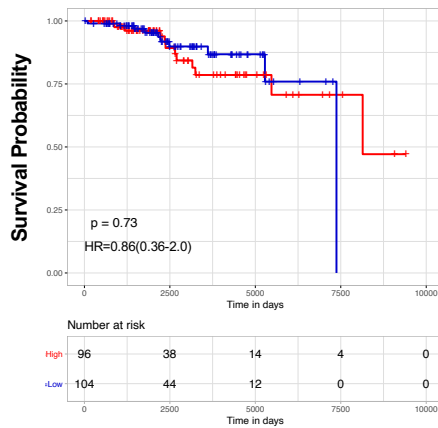

ID1

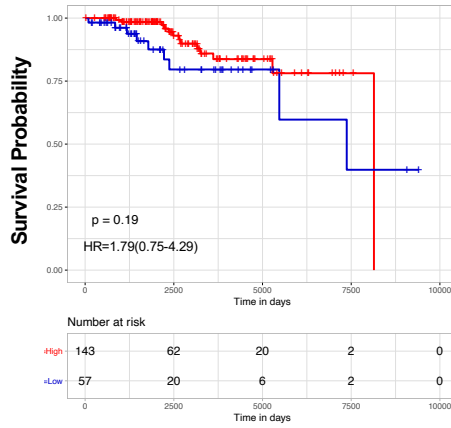

UNC93B2

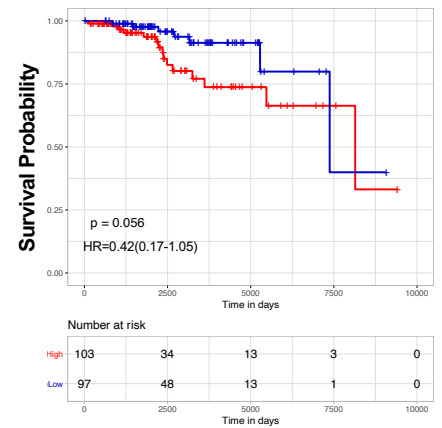

Black M7 Module

Brown M3 Module

Salmon M13 Module

Supplement: Supplementary file 16 — Additional file 16. Overall Survival of hub genes from networks associated with survival days. OS for nine hub genes from the M3, M7, and M13 networks were evaluated. The red lines denote OS of patients with high gene expression and the blue lines refer to patients with low expression. [file 12920_2021_1012_MOESM16_ESM.pdf]

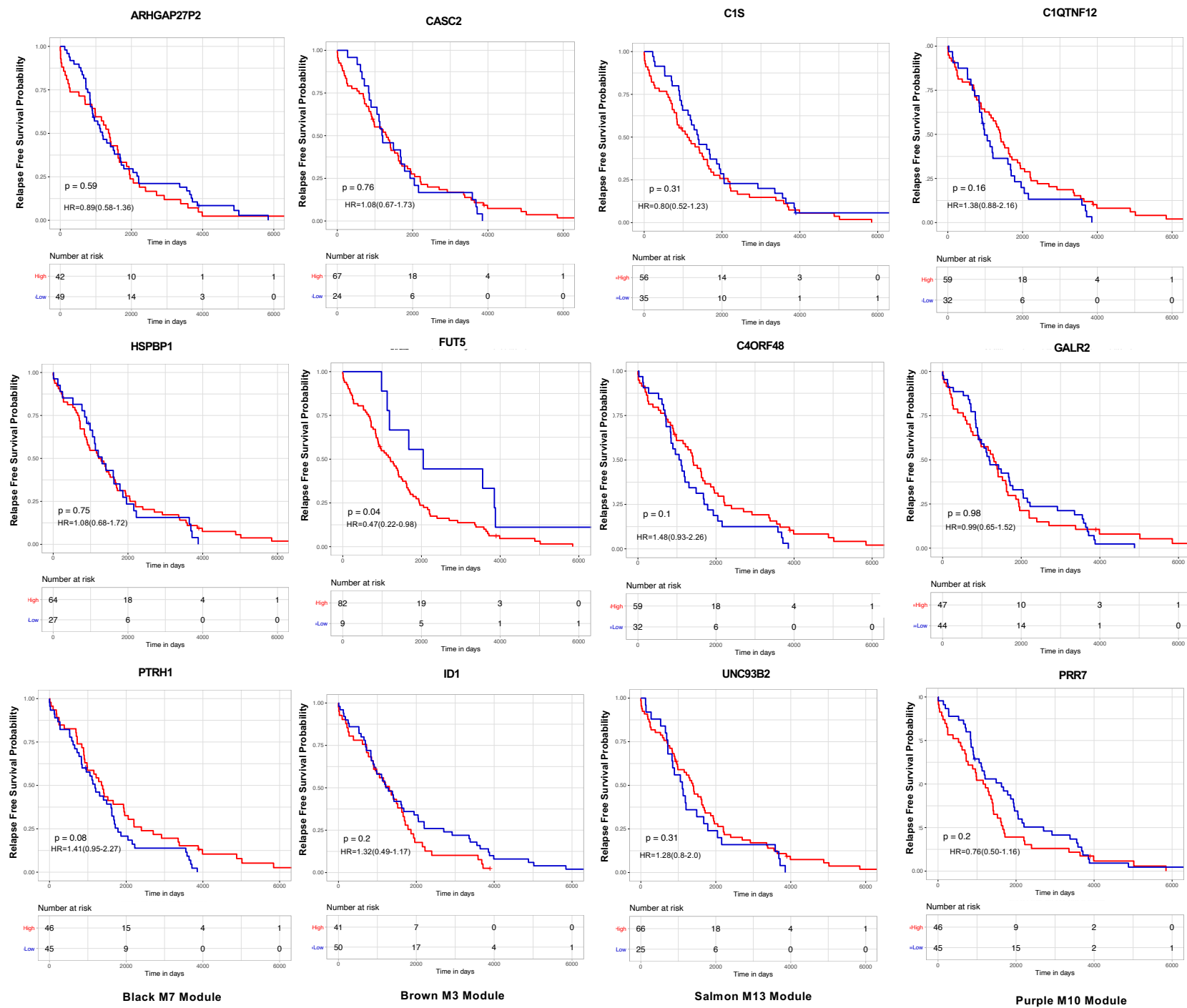

Supplement: Supplementary file 17 — Additional file 17. Relapse Free Survival of hub genes from Relapse associated networks. RFS for twelve hub genes from the M3, M7, M10, and M13 networks were evaluated. The red lines denote RFS of patients with high gene expression and the blue lines refer to patients with low expression. [file 12920_2021_1012_MOESM17_ESM.pdf]
