# Supplementary material for: Multivariate transcriptome analysis identifies networks and key drivers of chronic lymphocytic leukemia relapse risk and patient survival
Source: BMC Med Genomics. 2021 Jun 29;14:171. doi: 10.1186/s12920-021-01012-y (PMC8243588; doi:10.1186/s12920-021-01012-y)
Supplement: Supplementary file 5 — Additional file 5. Module Preservation of Modules based on Regression and IGHV Status. WGCNA’s Module Preservation analysis was applied according to regression (A, B) and IGHV (C, D) status. Median rank (left figure, y-axis) of modules determined by module preservation scores (right figure, y-axis) are displayed. Modules ranked closest to zero are the most preserved. The blue and red dotted lines denote cutoffs for a –log rank p value of 0.05 (blue) and p value < 0.00001. Module gene correlations are preserved regardless of IGHV and Regression status. [file 12920_2021_1012_MOESM5_ESM.pdf]

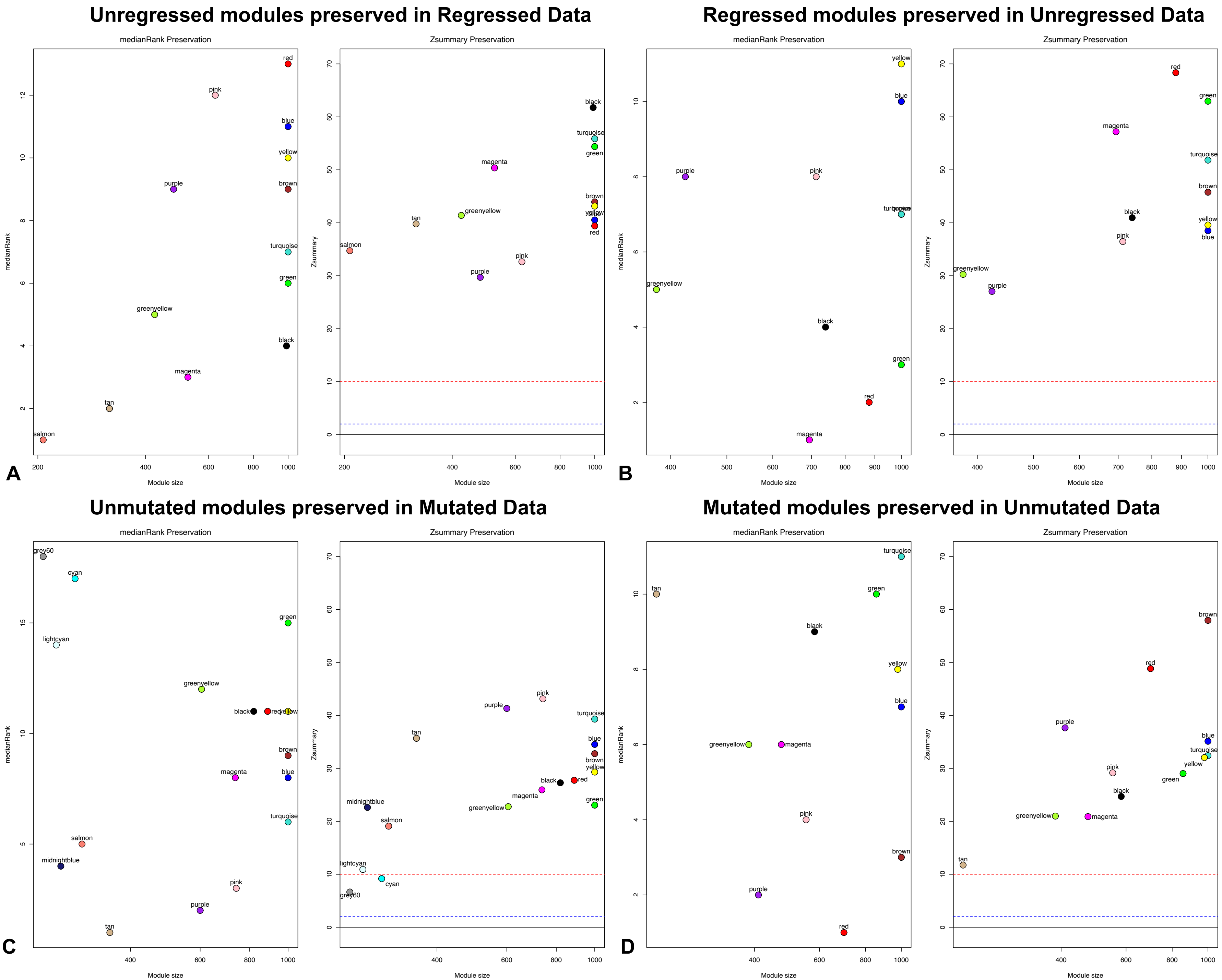
Unregressed modules preserved in Regressed Data
Regressed modules preserved in Unregressed Data
Unmutated modules preserved in Mutated Data
Mutated modules preserved in Unmutated Data
medianRank Preservation
Zsummary Preservation
medianRank
Zsummary
Module size
A
B
C
D
red
pink
blue
yellow
purple
brown
turquoise
green
greenyellow
black
magenta
tan
salmon
grey60
cyan
lightcyan
midnightblue
