# Supplementary material for: Multivariate transcriptome analysis identifies networks and key drivers of chronic lymphocytic leukemia relapse risk and patient survival
Source: BMC Med Genomics. 2021 Jun 29;14:171. doi: 10.1186/s12920-021-01012-y (PMC8243588; doi:10.1186/s12920-021-01012-y)
Supplement: Supplementary file 6 — Additional file 6. Module-Trait Heatmaps of Regressed and Unregressed ICGC Data (.pdf). Modules for unregressed (A) and regressed (B, C) datasets are denoted on the x-axis, traits on y-axis. B Modules-trait relationships following regression of sex and age. C reprEsents modules-trait relationships following regression of sex, age, and IGHV status. The blue-white-red colors inside of the heatmap indicate positive (red), negative (blue), and no(white) correlations. The numbers, inside the heatmaps, represent the correlation test p values. WGCNA of unregressed data produced more modules (13 vs 11) and significant module-trait relationships (45 vs 34) than the regressed (panel B) dataset. [file 12920_2021_1012_MOESM6_ESM.pdf]

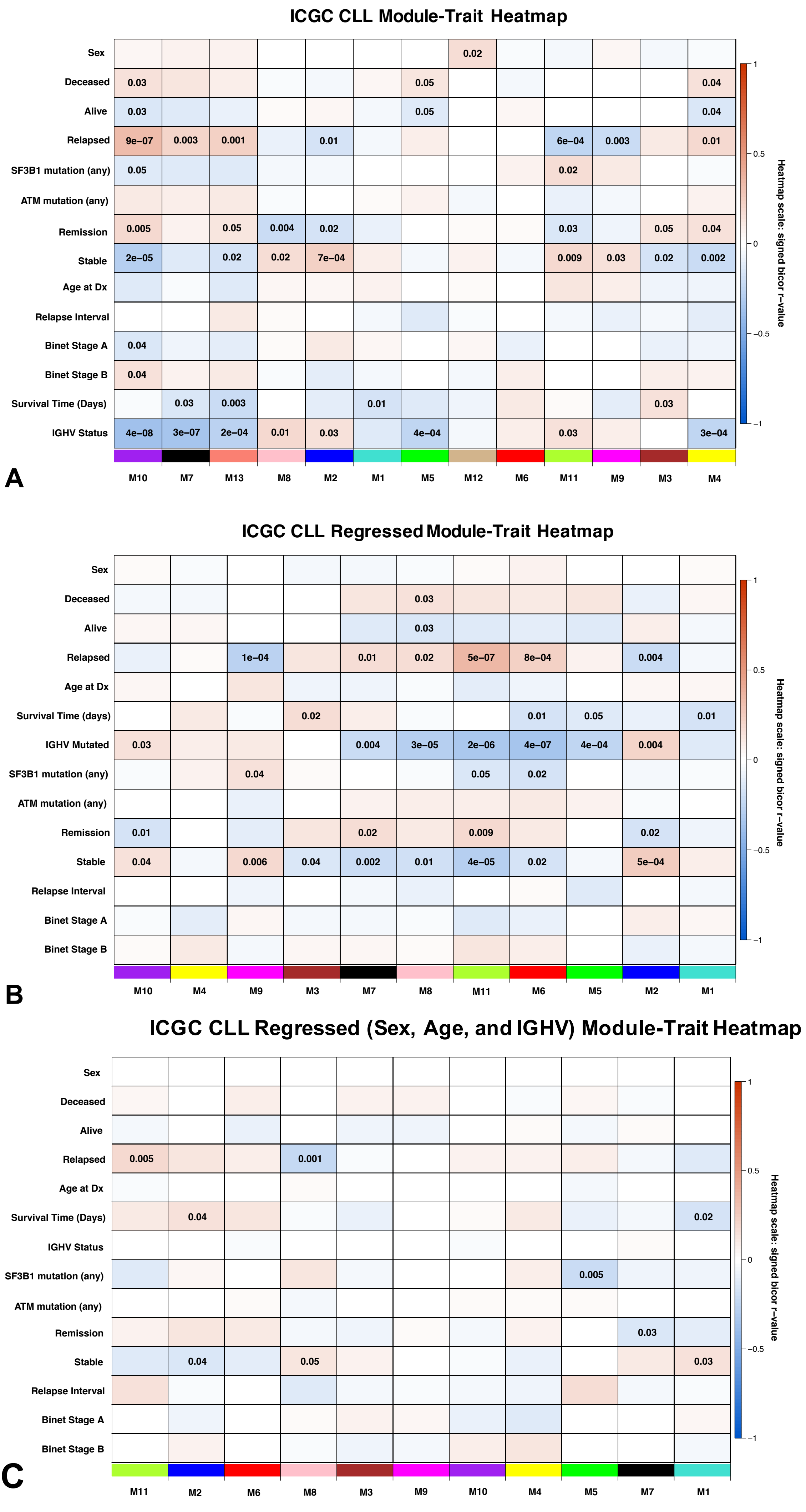

ICGC CLL Module-Trait Heatmap
Sex
Deceased
Alive
Relapsed
SF3B1 mutation (any)
ATM mutation (any)
Remission
Stable
Age at Dx
Relapse Interval
Binet Stage A
Binet Stage B
Survival Time (Days)
IGHV Status
0.02
0.03
0.05
0.04
0.03
0.05
0.04
9e−07
0.003
0.001
0.01
6e−04
0.003
0.01
0.05
0.02
0.005
0.05
0.004
0.02
0.03
0.05
0.04
2e−05
0.02
0.02
7e−04
0.009
0.03
0.02
0.002
0.04
0.04
0.03
0.003
0.01
0.03
4e−08
3e−07
2e−04
0.01
0.03
4e−04
0.03
3e−04
M10
M7
M13
M8
M2
M1
M5
M12
M6
M11
M9
M3
M4
A
Heatmap scale: signed bicor r−value
1
0.5
0
−0.5
−1
ICGC CLL Regressed Module-Trait Heatmap
Sex
Deceased
Alive
Relapsed
Age at Dx
Survival Time (days)
IGHV Mutated
SF3B1 mutation (any)
ATM mutation (any)
Remission
Stable
Relapse Interval
Binet Stage A
Binet Stage B
0.03
0.03
1e−04
0.01
0.02
5e−07
8e−04
0.004
0.02
0.01
0.05
0.01
0.03
0.004
3e−05
2e−06
4e−07
4e−04
0.004
0.04
0.05
0.02
0.01
0.02
0.009
0.02
0.04
0.006
0.04
0.002
0.01
4e−05
0.02
5e−04
M10
M4
M9
M3
M7
M8
M11
M6
M5
M2
M1
B
Heatmap scale: signed bicor r−value
1
0.5
0
−0.5
−1
ICGC CLL Regressed (Sex, Age, and IGHV) Module-Trait Heatmap
Sex
Deceased
Alive
Relapsed
Age at Dx
Survival Time (Days)
IGHV Status
SF3B1 mutation (any)
ATM mutation (any)
Remission
Stable
Relapse Interval
Binet Stage A
Binet Stage B
0.005
0.001
0.04
0.02
0.005
0.03
0.04
0.05
0.03
M11
M2
M6
M8
M3
M9
M10
M4
M5
M7
M1
C
Heatmap scale: signed bicor r−value
1
0.5
0
−0.5
−1
