# Supplementary material for: Multivariate transcriptome analysis identifies networks and key drivers of chronic lymphocytic leukemia relapse risk and patient survival
Source: BMC Med Genomics. 2021 Jun 29;14:171. doi: 10.1186/s12920-021-01012-y (PMC8243588; doi:10.1186/s12920-021-01012-y)
Supplement: Supplementary file 13 — Additional file 13. Optimum RFS Gene Combination AUC in U-CLL patients. Receiver Operator curves for Optimum biomarker gene combinations are shown for each time point: 15 months (A), 18 months (B), 3 years (C), and 5 years (D), The y-axis represents the percentage of patients who were true positives for relapse, whereas the x-axis represents the percentage of patients who were true negatives. The AUC (top left legend) for each time point is represented by a distinct color: dark orange (15 months), red (18 months), dark red (3 years), and magenta (5 years). [file 12920_2021_1012_MOESM13_ESM.pdf]

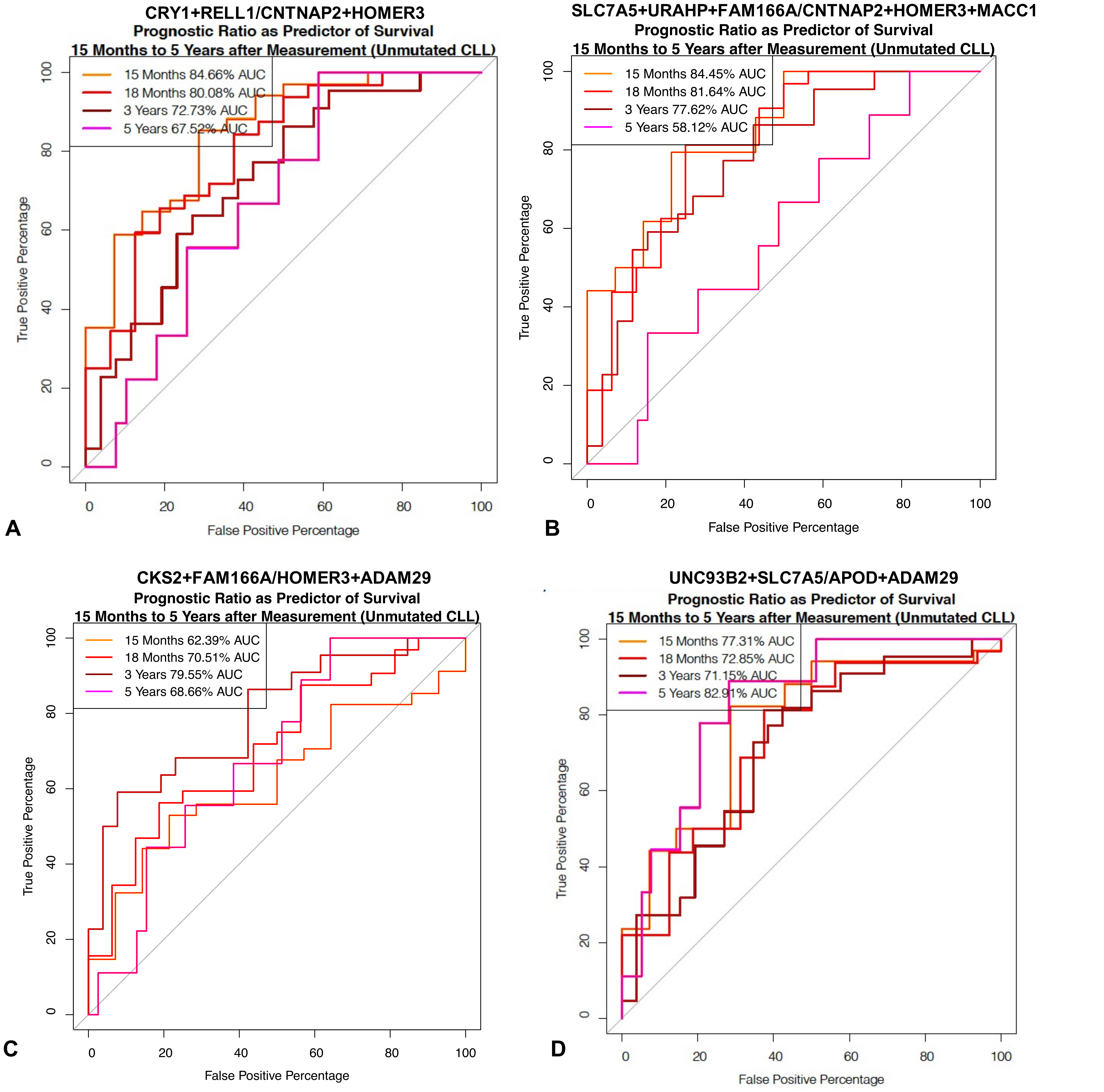

CRY1+RELL1/CNTNAP2+HOMER3
Prognostic Ratio as Predictor of Survival
15 Months to 5 Years after Measurement (Unmutated CLL)
15 Months 84.66% AUC
18 Months 80.08% AUC
3 Years 72.73% AUC
5 Years 67.52% AUC
True Positive Percentage
False Positive Percentage
0
20
40
60
80
100
A
SLC7A5+URAHP+FAM166A/CNTNAP2+HOMER3+MACC1
Prognostic Ratio as Predictor of Survival
15 Months to 5 Years after Measurement (Unmutated CLL)
15 Months 84.45% AUC
18 Months 81.64% AUC
3 Years 77.62% AUC
5 Years 58.12% AUC
True Positive Percentage
False Positive Percentage
B
CKS2+FAM166A/HOMER3+ADAM29
Prognostic Ratio as Predictor of Survival
15 Months to 5 Years after Measurement (Unmutated CLL)
15 Months 62.39% AUC
18 Months 70.51% AUC
3 Years 79.55% AUC
5 Years 68.66% AUC
True Positive Percentage
False Positive Percentage
C
UNC93B2+SLC7A5/APOD+ADAM29
Prognostic Ratio as Predictor of Survival
15 Months to 5 Years after Measurement (Unmutated CLL)
15 Months 77.31% AUC
18 Months 72.85% AUC
3 Years 71.15% AUC
5 Years 82.91% AUC
True Positive Percentage
False Positive Percentage
D
